# Bridging a curriculum gap: a structured model for integrating head and neck ultrasound training into undergraduate dental education

**DOI:** 10.1186/s12909-025-08521-9

**Published:** 2026-01-07

**Authors:** Johannes Weimer, Alexa Lippe, Marie Brandt, Maximilian Rink, Lisa Morlock, Julian Künzel, Luisa Symeou, Liv Weimer, Christoph Sproll, Holger Buggenhagen, Lukas Müller, Lukas Pillong, Moritz Knebel, Marie Stäuber, Rainer Mengel, Oliver Kripfgans, Julia Weinmann-Menke, Anke Hollinderbäumer, Florian Recker, Bilal Al-Nawas

**Affiliations:** 1 Rudolf Frey Learning Clinic, University Medical Centre of the Johannes Gutenberg University Mainz, Langenbeckstraße 1, Mainz, 55131 Germany; 2 Department of Medicine I and Center for Immunotherapy, University Medical Centre of the Johannes Gutenberg University Mainz, Mainz, Germany; 3 Department of Oral and Maxillofacial Surgery - Facial Plastic Surgery, University Medical Center of the Johannes Gutenberg-University of Mainz, Mainz, Germany; 4 Department of Otorhinolaryngology, Brothers of Mercy Hospital St. Elisabeth, Straubing, Germany; 5 Department of Otorhinolaryngology, Head and Neck Surgery, University Hospital Regensburg, Regensburg, Germany; 6 Department of Periodontology and Operative Dentistry, University Medical Center of the Johannes Gutenberg University Mainz, Mainz, Germany; 7 Department of Radiation Oncology and Radiotherapy, University Medical Center of the Johannes Gutenberg University Mainz, Mainz, Germany; 8 Department of Oral and Maxillofacial Surgery, University Hospital Düsseldorf, Heinrich-Heine-University Düsseldorf, Düsseldorf, Germany; 9 Department of Diagnostic and Interventional Radiology, Mainz University Hospital, Mainz, Germany; 10 Department for Otorhinolaryngology and Head- and Neck-Surgery, Saarland University Medical Center, Homburg, Germany; 11 Department of Prosthetic Dentistry, School of Dental Medicine, Philipps-University, Marburg, Lahn Germany; 12 Department of Radiology, Medical School, University of Michigan, Ann Arbor, USA; 13 Department of Obstetrics, University Hospital Cologne, Cologne, Germany

**Keywords:** Dental education, Ultrasonography, Blended learning, Curriculum development, ICAP framework, Kern’s six-step approach, Competency-based training

## Abstract

**Introduction:**

Ultrasonography is increasingly relevant in dental and maxillofacial diagnostics, yet structured training opportunities for dental students remain scarce. This study aimed to develop, implement, evaluate and validate a blended learning head and neck ultrasound (HNUS) curriculum for dental students, guided by the ICAP (Interactive, Constructive, Active, Passive) framework and Kirkpatrick’s evaluation model.

**Material and methods:**

Following Kern’s six-step approach to curriculum development, a prospective quasi-experimental design was applied. Dental students (study group) completed the curriculum and were assessed at three time points (T1: pre-course; T2: Immediate post-course; T3: three-month follow-up). Physicians with prior certified ultrasound training served as a reference group at T2. The intervention combined video-based e-learning modules, lecture notes, anatomy posters, and peer-assisted hands-on training. Primary outcomes were objective knowledge (theory test at T1-T3) and practical performance (Direct Observation of Procedural Skills = DOPS at T2). Secondary outcomes included self-assessed competence, satisfaction, and attitudes toward ultrasound education (7-point Likert scale).

**Results:**

A total of 64 students completed T1–T2, and 21 completed T3 three months after course completion. At T2, students demonstrated significant gains in theoretical knowledge compared to T1 (*p* < 0.001, d = − 4.1). Although a moderate decline was observed at T3, scores remained substantially above T1 (*p* < 0.001). In the direct comparison at T2, physicians achieved significantly higher theory test scores than students (*p* = 0.011). While overall DOPS performance did not differ significantly between students and physicians (*p* = 0.59), domain-specific variations were observed. Self-assessed competencies increased markedly from T1 to T2 (*p* < 0.0001), with sustained improvements observed at the three-month follow-up (T3) in the subgroup of participants who completed all assessments Evaluation of curricular components yielded consistently high ratings (mean 6.1–6.5/7), with e-learning and hands-on stations most valued. Students strongly endorsed the integration of ultrasonography into dental curricula and favoured blended learning approaches.

**Conclusion:**

This study provides initial evidence that a structured, ICAP-informed, and Kern’s six-step–guided blended HNUS curriculum for dental students is feasible, effective, and well accepted. Students achieved substantial improvements in theoretical knowledge, practical skills, and self-perceived competencies, approximating physicians’ performance levels overall, with some domain-specific differences. The findings support the curricular integration of ultrasonography into undergraduate dental education.

**Supplementary Information:**

The online version contains supplementary material available at 10.1186/s12909-025-08521-9.

## Introduction

### Background

Imaging procedures such as X-rays, magnetic resonance imaging (MRI), and computer tomography (CT) scans are integral to everyday dental care, whether in diagnosis, monitoring therapeutic success, or as part of the actual treatment [1, 2]. While three-dimensional imaging with CT or cone beam CT (DVT) is widely used in implantology and fracture examination, classic two-dimensional radiographs remain the standard in areas such as endodontics and caries diagnosis [1–3]. By contrast, the main advantage of magnetic resonance imaging (MRI) lies in its superior visualisation of soft tissue structures [1, 4].

Alongside MRI, ultrasonography has emerged as another modality that extends diagnostic possibilities beyond hard tissue imaging. Ultrasonography represents an essential diagnostic tool in oral and maxillofacial surgery (OMFS) and is gaining importance in dentistry as a complementary imaging technique [5–16]. Although ultrasonography is well established across most medical specialties, its role in OMFS and dentistry has expanded with the development of ultra-high frequency transducers. It now offers a valuable option for imaging peri-implantitis, evaluating and differentiating soft tissue lesions such as cysts, abscesses, tumours, fractures, temporomandibular joint dysfunction and even hard tissue pathologies including caries, bone and tooth fractures and root defects [5–14, 17–19].

With ultrasonography’s growing relevance in patient care, the training of dental students in sonography is increasingly important [20]. For medical students, structured ultrasound courses of varying scope are already well established at many institutions worldwide, but dentistry has not yet reached the same level of integration [21, 22]. This discrepancy reflects broader structural challenges in dental education, including limited adoption of new diagnostic technologies, insufficient faculty training, and lack of standardisation across institutions, as demonstrated in a recent scoping review [23]. Previous studies have shown that sonography training can result in a wide range of benefits, including improved anatomical skills [24–26], a better understanding of the advantages and disadvantages of different imaging techniques [17, 24, 27], and enhanced interdisciplinary exchange [28]. Some of these benefits have also been demonstrated specifically in the context of dental ultrasound education [22].

Although medical education has seen substantial curricular innovation in response to technological and diagnostic advances, dental curricula have been slower to adapt [29]. In medicine, structured pathways for acquiring skills in head and neck sonography are already well established, both for students [30–35] and for doctors who are already licensed [5, 36–42]. Demand for such training is high [43]. Dentistry, however, has not kept pace with this development, and structured training approaches to head and neck ultrasonography in dental education remain underdeveloped [22, 44]. This lag is inconsistent with the modality’s increasing clinical importance.

Reflecting this lack of structured training opportunities, a recent prospective study involving dentistry students from higher semesters revealed that most of the participants had never performed a head and neck ultrasound. At the same time, students expressed a strong interest in ultrasound training, with particularly high demand for modules on the temporomandibular joint; teeth, roots, and alveolar processes; and salivary gland sonography. These findings underline the urgent need to develop and implement a structured, practice-oriented head and neck ultrasound curriculum tailored to dentistry students’ needs [20].

Despite the clinical relevance of ultrasonography in dental and maxillofacial diagnostics [45]—and the clear demand from students and experts for structured training—this vital imaging modality has not been standardised or systematically integrated into undergraduate dental curricula, either in Germany or internationally. This gap in dental education represents a missed opportunity to enhance diagnostic skills and patient care. Addressing this deficit is essential to expand the diagnostic capabilities of future dentists and to ensure high-quality, practice-oriented training. This need is particularly underscored by the national competency-based learning objective frameworks for medical and dental education (NKLM, NKLZ), which emphasise the importance of diagnostic imaging, including ultrasonography, in dental curricula [44, 46].

Although multiple reports have highlighted the clinical relevance of ultrasonography in dentistry, its adoption in undergraduate curricula has been limited by structural, organisational, and educational barriers. Demonstrating that dental students can successfully acquire ultrasound skills through a structured, competency-based curriculum may help address these challenges by providing a feasible and transferable model for curricular integration.

Recent models in learning psychology emphasise that the depth of cognitive engagement is a key determinant of successful knowledge acquisition and skill development. The ICAP (Interactive, Constructive, Active, Passive) framework categorises learner activities according to their cognitive demand, proposing that learning outcomes increase with the level of engagement [47]. Building on this framework, the present study evaluated the feasibility, effectiveness, and acceptance of a newly developed head and neck ultrasound curriculum for dental students. The curriculum was delivered in a blended learning format and its evaluation was conceptually aligned with Kirkpatrick’s four levels of training outcomes [48].

The central research question was: What effects does an ICAP-informed blended learning curriculum have on the development of sonographic competencies in dental students—encompassing knowledge, practical skills, self-assessment, and attitudes—compared to baseline and to a clinically experienced reference group, and to what extent can the curriculum be evaluated and validated using the Kirkpatrick model? We hypothesised that a systematically developed, ICAP-informed blended learning curriculum for head and neck ultrasound in dentistry would (1) significantly enhance students’ theoretical knowledge and practical ultrasound competencies compared to baseline; (2) lead to measurable improvements in self-perceived confidence, motivation, and satisfaction; and (3) enable dental students to achieve performance levels in fundamental ultrasound skills comparable to those of a physician reference group. Collectively, these outcomes would provide evidence of the curriculum’s validity within the Kirkpatrick model and support its feasibility for curricular integration.

## Methods

### Study design and participants

**Fig. 1:**
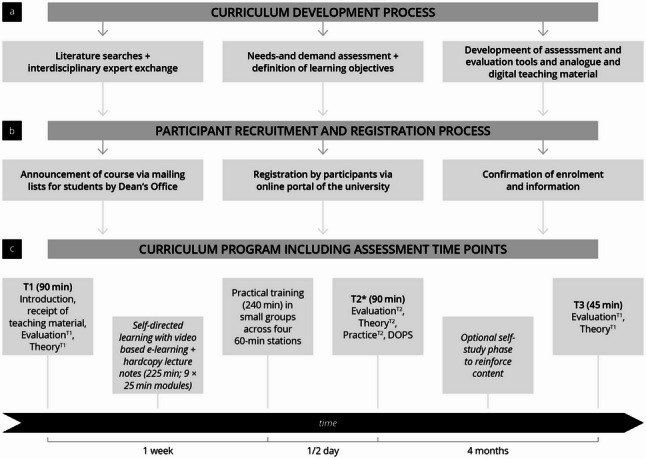
Study planning, study process, and curriculum implementation. DOPS = Direct Observation of Procedural Skills Test. **a** Curriculum development process; **b** Timeline of participant recruitment; **c** Curriculum Implementation including assessments. *These evaluations and tests were also performed by the reference group

This prospective quasi-experimental educational study (Fig. 1) was conducted at a German university hospital between 2024 and 2025 and documented in accordance with the STROBE (Strengthening the Reporting of Observational Studies in Epidemiology) guidelines [49]. The curriculum was developed following Kern´s Six-Step Approach to medical education [50], grounded in a previous needs assessment [20], and informed by the ICAP framework [47]. Its components—including instructional content, teaching materials, evaluation tools, and assessment formats—were designed and refined through expert consensus by a multidisciplinary team from the fields of otorhinolaryngology, oral and maxillofacial surgery, radiology, dentistry, and medical education.

The study employed a mixed longitudinal and cross-sectional design, assessing dental students at three time points (T1–T3) and including a physician reference group at T2 to provide a professional benchmark for curriculum validation. At each time point, participants completed evaluations (including baseline assessment at T1) and written theoretical tests, with an additional practical examination conducted at T2 [51–56]. The assessments were designed to capture both objective competencies as the primary outcome and, as secondary outcomes, perceived competency gains and participant satisfaction with the curriculum, instructional materials, and teaching methods, as well as attitudes toward ultrasound education and instructional formats as informed by established frameworks and prior literature [21, 33, 51, 55, 57–60].

To evaluate the curriculum’s effectiveness, the assessment strategy was aligned with Kirkpatrick’s four levels of training outcomes [48, 61]. The primary focus was on Level 1 (Reaction), capturing participants’ satisfaction and feedback on the learning experience, and Level 2 (Learning), measuring gains in theoretical knowledge and practical ultrasound skills through written examinations and structured performance assessments. Preliminary insights into Level 3 (Behaviour) were obtained by measuring students’ self-reported confidence and perceived readiness to apply the acquired ultrasound skills in clinical settings. Direct evaluation of sustained behavioural change or patient-related outcomes (Level 4, Results) was beyond the scope of this study.

Dental students from the first clinical semester onward were eligible to participate voluntarily by registering via an online platform after completing the pre-clinical phase of their studies. All students in the clinical semesters were informed about the course and the registration process through official university communication channels. Inclusion in the study required full participation in the course, completion of all evaluation and assessment instruments, and provision of informed consent. Participation status had no impact on students’ progression within the compulsory curriculum and carried no academic advantages or disadvantages. Students were excluded if data sets were incomplete or informed consent was withdrawn.

The reference cohort, serving as a control group, comprised medical doctors enrolled in certified head and neck ultrasound (HNUS) [62] courses who participated in at least one of the objective assessment (theory test and/or DOPS) formats at T2 and provided baseline data.

### Curriculum design, learning objectives, teaching material and teachers

The curriculum was developed based on a needs and demand analysis, aligned with national competency frameworks and the educational content of the German Society for Ultrasound in Medicine (DEGUM) head and neck ultrasound guidelines, and finalised through expert consensus [20, 44, 62]. A complete list of learning objectives is provided in Supplement 1. The educational intervention (Fig. 1) was designed as a blended learning curriculum combining asynchronous, video-based e-learning with structured hands-on training [59, 63, 64]. Informed by the ICAP framework [47], the curriculum combined self-directed e-learning (constructive engagement) modules with supervised, peer-based hands-on sessions (interactive), to promote theoretical knowledge acquisition and practical ultrasound competence. After an introductory session (45 min) and initial assessment at T1 (45 min), participants were provided with e-learning modules and printed lecture notes to support self-directed preparation for the practical training (225 min) and post-course consolidation.

The e-learning component comprised nine video modules (approx. 25 min each) covering relevant anatomical regions and sonographic techniques in a structured and clinically oriented manner. Module 0 introduced clinical ultrasound, its historical development, and fundamental physical principles. Modules 1 to 8 focused on specific anatomical regions: floor of the mouth; neck levels; submandibular space and tonsils; parotid gland; dental structures and implants; temporomandibular joint and masticatory muscles; bony landmarks; and intraoral ultrasound of the tongue and tonsils. Each module (except for Module 0) followed a standardised didactic structure with defined learning objectives, anatomical orientation, scanning technique and sonoanatomy, a brief pathology overview, and a clinical take-home message. The modules were developed in-house by experts in sonography, oral and maxillofacial surgery, otorhinolaryngology, radiology, and medical education/didactics.

The practical component of the course (240 min) comprised four instructor-led ultrasound stations (60 min each) covering: (1) neck levels; (2) salivary glands and floor of the mouth; (3) bony facial structures and temporomandibular joint; and (4) intraoral ultrasonography of teeth, implants, tongue, and tonsils. These stations were directly aligned with the e-learning content to promote integrated, application-oriented learning. Anatomy posters were also used during the practical sessions. To ensure instructional consistency and alignment with the defined learning objectives, all tutors received standardised station guides outlining the specific content to be taught. Upon completion of the practical sessions, participants completed the T2 assessment phase (90 min). The curriculum primarily covered normal head and neck anatomy and basic sonographic techniques, with pathological findings included only for illustration.

### Assessment tools

The evaluation and assessment formats were developed in alignment with the predefined curricular content and methodologically guided by current recommendations and previous work [33, 41, 51–56, 59, 64]. Their design was carried out by an interdisciplinary team of experts in medical education, otorhinolaryngology, oral and maxillofacial surgery, dentistry, and radiology.

#### Evaluations (T1–T3) — Kirkpatrick level 1 and level 3 (self-reported)

At T1, baseline data were collected, including demographic data, academic background, and prior experience with head and neck ultrasound or other imaging techniques. At T2, participants additionally reported on their use of the provided learning materials for self-directed preparation and any new prior experience. Across all time points, questionnaires assessed participants’ motivation, interest in relevant specialties and imaging modalities, and their attitudes toward ultrasound education and its curricular integration. Self-assessed competencies were also gathered at all three time points, covering specific ultrasound skills, normal findings/sonoanatomy, and assessment of pathologies. At T2, participants further evaluated specific curricular elements, including the e-learning modules, printed lecture notes, educational posters, practical stations, and overall course implementation. The instruments included both 7-point Likert-scale items (1 = strongly disagree/very unsure; 7 = strongly agree/very sure) and open-ended questions. The completion time for the evaluations was approximately 10 min at each measurement point. The original evaluation instruments are provided in Supplement 2.

#### Theory tests (T1–T3) — Kirkpatrick level 2 (Learning)

The tests were administered in paper-based format with a completion time of 35 min. The test relied primarily on open-ended responses to support active recall and deeper cognitive processing and knowledge transfer, consistent with the Constructive level of the ICAP framework, which emphasizes active generation and elaboration of knowledge [47, 53, 55]. The content covered five core domains: anatomical basics (12 points); ultrasound basics including artifacts, terminology, and technical parameters (25 points); image-based assignment tasks requiring the correct allocation of transducer positions and sectional planes (4 points): labelling of sonoanatomy in normal findings (27 points); and labelling of pathological findings (11 points); amounting to a total of 79 achievable points. The original test administered at each time point is provided in Supplement 3. All participant responses were anonymised and compiled for centralised scoring. To ensure subject-matter accuracy and content validity, six independent experts with advanced ultrasound qualifications (DEGUM level II–III) reviewed all answers. Each expert independently identified the responses they considered correct, and a response was awarded one point if at least four out of six experts (≥ 66%) deemed it correct. Responses endorsed by three or fewer experts were classified as incorrect, and no additional consensus round was conducted. This consensus-based scoring approach provided a robust and discipline-specific standard for assessing open-ended items [65, 66].

#### Direct observation of procedural skills (DOPS) test (T2) — Kirkpatrick level 2 (Learning)

Practical ultrasound skills were assessed using a structured DOPS format adapted for head and neck ultrasonography in dentistry [41, 51], specifically designed to capture key psychomotor components of ultrasound performance [67]. The examination was conducted at T2 by a trained examiner. Participants performed a standardised orientational ultrasound examination on a volunteer, including appropriate communication and demonstration of defined anatomical sectional planes. The assessment comprised seven domains with a maximum total score of 54 points: patient guidance and communication (8 points); transducer handling and image optimization (8 points); as well as correct plane adjustment and anatomical identification for the neck level (7 points); floor of the mouth (6 points); tonsillar/submandibular region (6.5 points); and parotid gland including temporomandibular joint (6.5 points). An overall performance rating (8 points) completed the evaluation. Each domain combined process-based criteria (e.g., coupling, orientation, image settings) and outcome-based criteria (e.g., anatomical structure identification). Grading followed a structured three-tier scale (correct, verbal prompt, manual assistance). Examiners were calibrated in advance and used a standardised checklist ( Supplement 4) to ensure comparability and objectivity.

### Data collection and statistics

Data were collected using a paper-based evaluation, theory-test, and practical exam, which were subsequently entered manually into Microsoft Excel (Version 16.0) for further processing. Statistical analyses were performed in RStudio (RStudio Team [2020]. RStudio: Integrated Development for R. RStudio, PBC, http://www.rstudio.com, last accessed on 04/08/2025) with R 4.0.3 (A Language and Environment for Statistical Computing, R Foundation for Statistical Computing, http://www.R-project.org; last accessed on 04/08/2025). Where applicable, main scale scores were calculated as the average of the subscale scores. Internal consistency of the self-assessment, evaluation, and theory/practical test scales was examined using Cronbach’s alpha and McDonald’s omega. Binary and categorical baseline variables are reported as absolute numbers and percentages. Descriptive statistics were used to summarise baseline characteristics and prior experience, with absolute and relative frequencies for categorical variables, and mean ± standard deviation (SD) or median with interquartile range (IQR) for continuous variables, depending on data distribution. Longitudinal comparisons across the three time points (T1, T2, T3) were conducted using repeated-measures ANOVA or the Kruskal-Wallis test, followed by appropriate pairwise post hoc tests (t-test or Mann-Whitney U). These tests were also used to calculate the influence of the factors on the subjective and objective results. Effect sizes were calculated using Cohen’s d. For all effect sizes, negative values indicate improvement or increase from earlier to later time points. Pairwise correlations between variables were analysed, including effect sizes and significance levels. P-values < 0.05 were considered statistically significant. A power analysis yielded a minimum sample size of 59 participants per group (study/control) for an effect size of 0.6, at a significance level of 0.05 and a desired power of 0.9. Violin plots were used to visualise score distributions. These plots combine elements of boxplots and density plots, illustrating the probability density of the data at different values. The width of each “violin” represents the frequency of participants achieving a given score, allowing a more detailed view of data spread and potential sub-distributions.

## Results

**Fig. 2:**
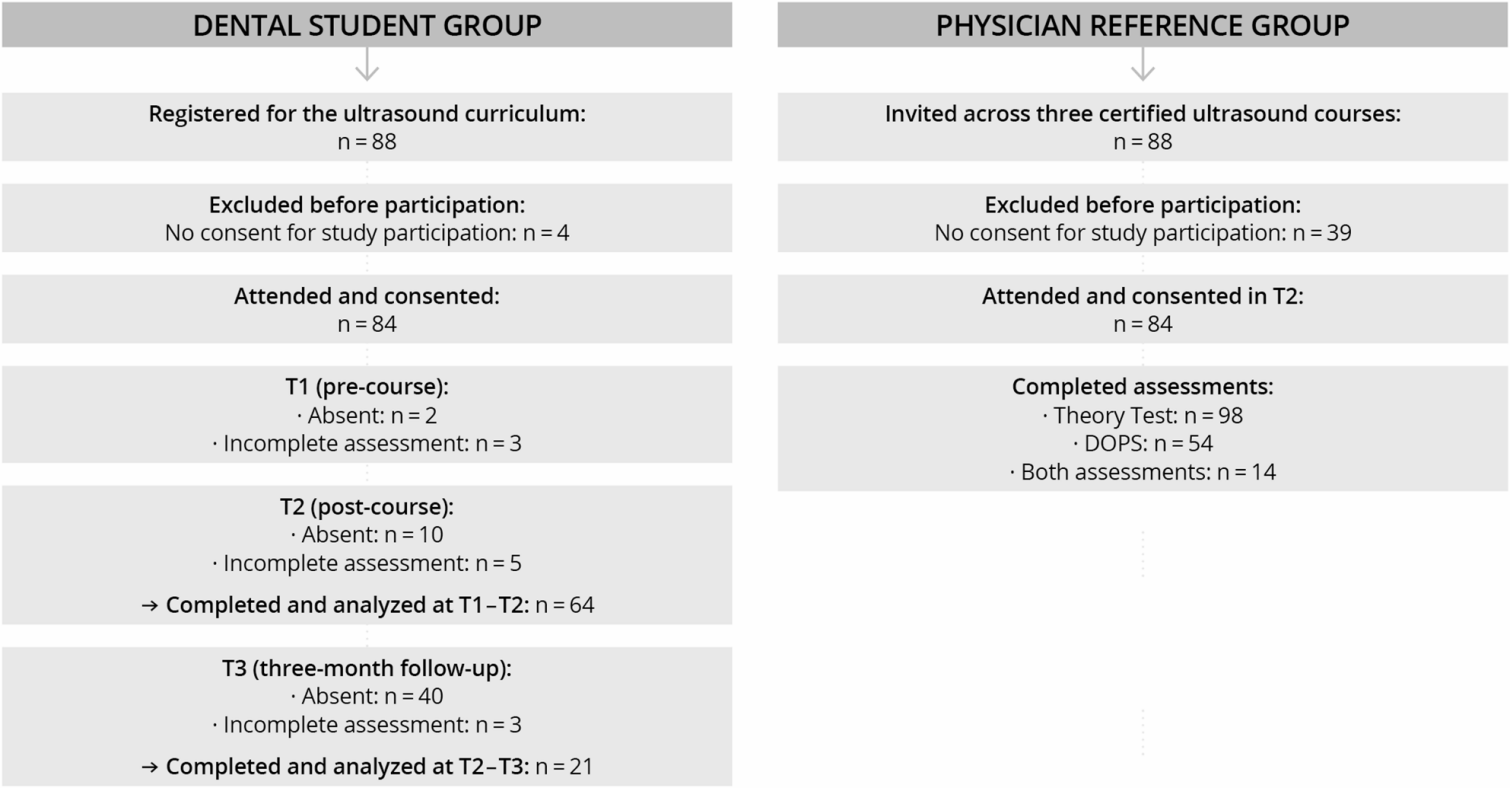
Participant flow diagram showing recruitment, inclusion, participation at each assessment time point (T1–T3), and final analysis numbers for the dental student cohort and the physician reference group

A total of 64 dental students completed the pre-course (T1) and post-course (T2) assessments, and 21 participated in the three-month follow-up (T3). The physician reference group comprised 141 unique participants, of whom 98 completed the theoretical assessment, 54 participated in the DOPS-based practical assessment, and 14 completed both. Accordingly, subgroup analyses are based on these respective samples(see Fig. 2).

### Baseline characteristics of the dental students

Most participants (see Supplement 5) were in the 8th or 9th semester (23.4% each) with a mean age of 23.9 ± 3.7 years; 71.9% were female. A total of 25.0% of participants reported previous vocational or academic training in a medical or dental-related field (e.g., dental technician, nursing, paramedic), but none had completed a prior degree in dentistry or medicine. 95.3% had completed anatomy and 67.2% radiology coursework. Only 10.9% had attended an otolaryngology lecture. Over 95% of participants had no prior experience with ultrasonography. Only one student reported previous attendance at an ultrasound course. None of the students had independently performed a head and neck ultrasound examination. Regarding preparatory behaviour, the majority of participants reported active use of the provided digital and print-based learning resources. Specifically, 87.3% of students used the e-learning platform for self-directed preparation, with a median usage time of 3 h (IQR: 2–4 h). The script-based lecture notes were used by 88.9% of participants, with a median usage time of 2 h (IQR: 0.5–3 h). Except for the modules on intraoral structures and bony landmarks, all e-learning chapters were completed by approximately 70% or more of the students.

### Baseline characteristics of the validation group

The group consisted predominantly of participants in postgraduate medical training (93.5% residents, 4.3% board-certified specialists, 2.2% senior physicians). The mean age was 30.9 ± 5.2 years, with a balanced gender distribution (50.0% male, 47.8% female). Most participants had a background in human medicine (82.4%), while 16.5% reported dual qualifications (dentistry and medicine). Regarding prior ultrasound experience, 40.2% had previously completed one or more ultrasound courses. Among those, 83.3% had received specific training in head and neck ultrasound. Participants reported having independently performed a median of 67.5 head and neck ultrasound examinations (range: 1–1200). A detailed overview of the baseline characteristics and prior ultrasonography exposure of the reference group is provided in Supplement 6.

### Data description

The reliability tests, as measured by Cronbach’s alpha and McDonald’s omega, indicated that the internal consistency of the main scales ranged from α = 0.77 to 0.98 and did not vary considerably. Minor deviations were observed for the theory test at T1 (α = 0.6) and the sonoanatomy subscale at T3 (α = 0.7).

### Results of the objective assessment (primary endpoints)

#### Theory test

**Fig. 3:**
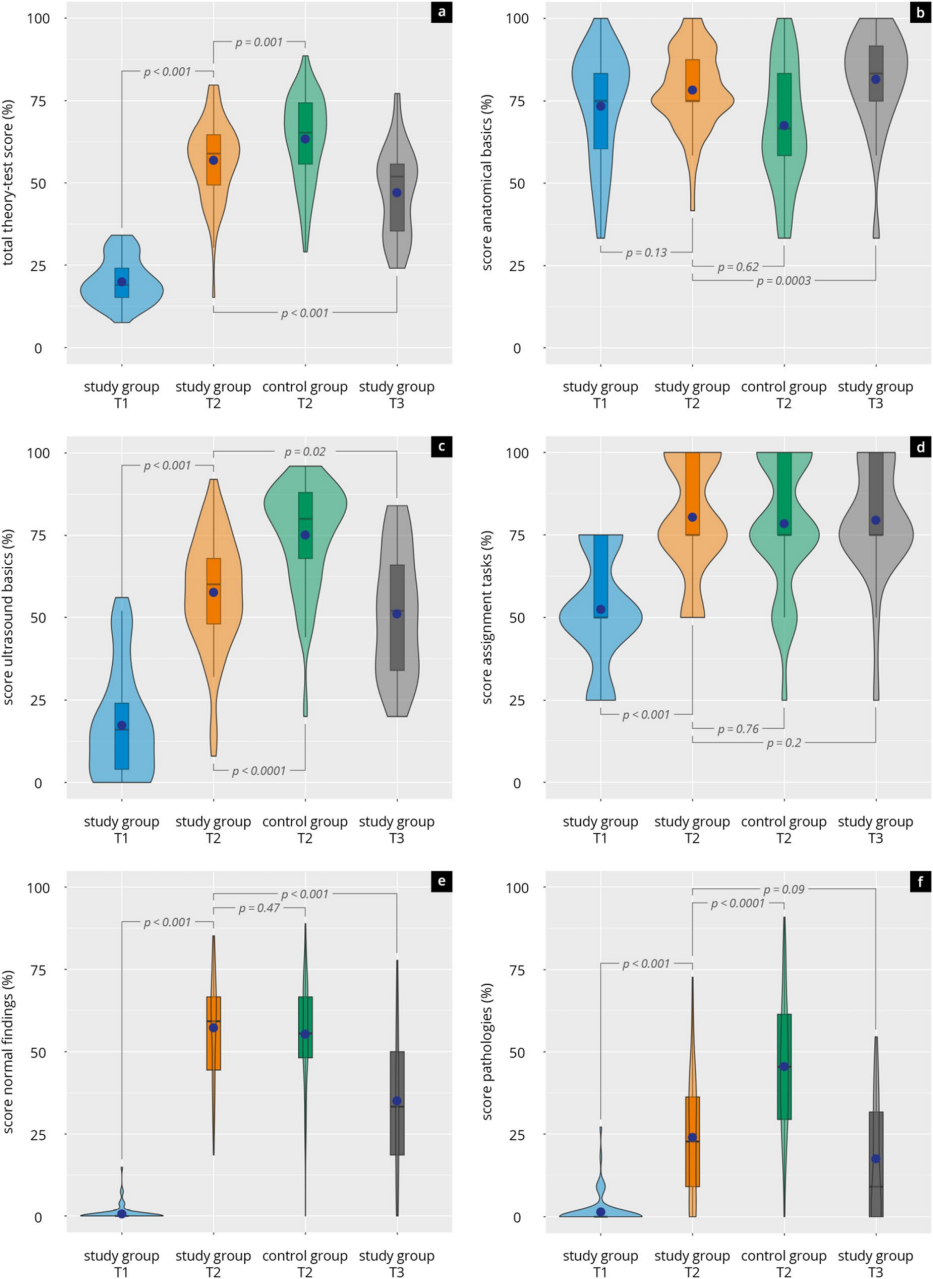
Results of the theory test in the study group (students) and the control group (physicians) at time points T1, T2, and T3. Shown are violin plots (with median [black bar], interquartile range, and mean [blue dot]) for (**a**) total score, **b** anatomical basics, **c** ultrasound basics, **d** assignment tasks, **e** normal findings, and (**f**) pathologies. Significance levels of group comparisons are indicated

The results of the theory test at time points T1–T3 are shown in Fig. 3 and Supplement 7.

Significance levels of group comparisons are indicated. Substantial improvements in theoretical knowledge were observed across all domains

From T1 to T2, participants showed highly significant gains in overall test performance (mean score increase: +36.3%, *p* < 0.001, d = − 4.1), with particularly large effects in the categories “normal findings” (d = − 5.2, *p* < 0.001), “ultrasound basics” (d = − 2.1, *p* < 0.001), “assignment task” (d= -1.8, *p* < 0.001), and “pathologies” (d = − 1.3, *p* < 0.001). Knowledge in anatomical basics also improved, though to a lesser extent (*p* = 0.13). Between T2 and T3, a moderate decline in total scores was observed (mean difference: − 12.8%, *p* < 0.001, d = − 1.05), though levels remained substantially above the scores of T1 (*p* < 0.01). The most significant (*p* < 0.01) drop was seen in the domain “normal findings”. The domain “pathologies” consistently showed the lowest scores at both T2 (24.2 ± 19.1%) and T3 (17.4 ± 18.2%), despite significant improvement from T1 (1.6 ± 4.7%). In contrast, scores in the “anatomical basics” domain continued to improve slightly from T2 to T3.

In comparison with the reference group, distinct differences emerged in specific knowledge domains of the theory test: physicians achieved significantly higher total scores (*p* < 0.001) and a higher score in “ultrasound basics” (*p* < 0.0001) and “pathologies” (*p* < 0.0001). In contrast, students outperformed physicians in “anatomical basics” (*p* = 0.0003). No significant differences were found in “normal findings” (*p* = 0.47) and “assignment tasks” (*p* = 0.76).

#### DOPS

**Fig. 4:**
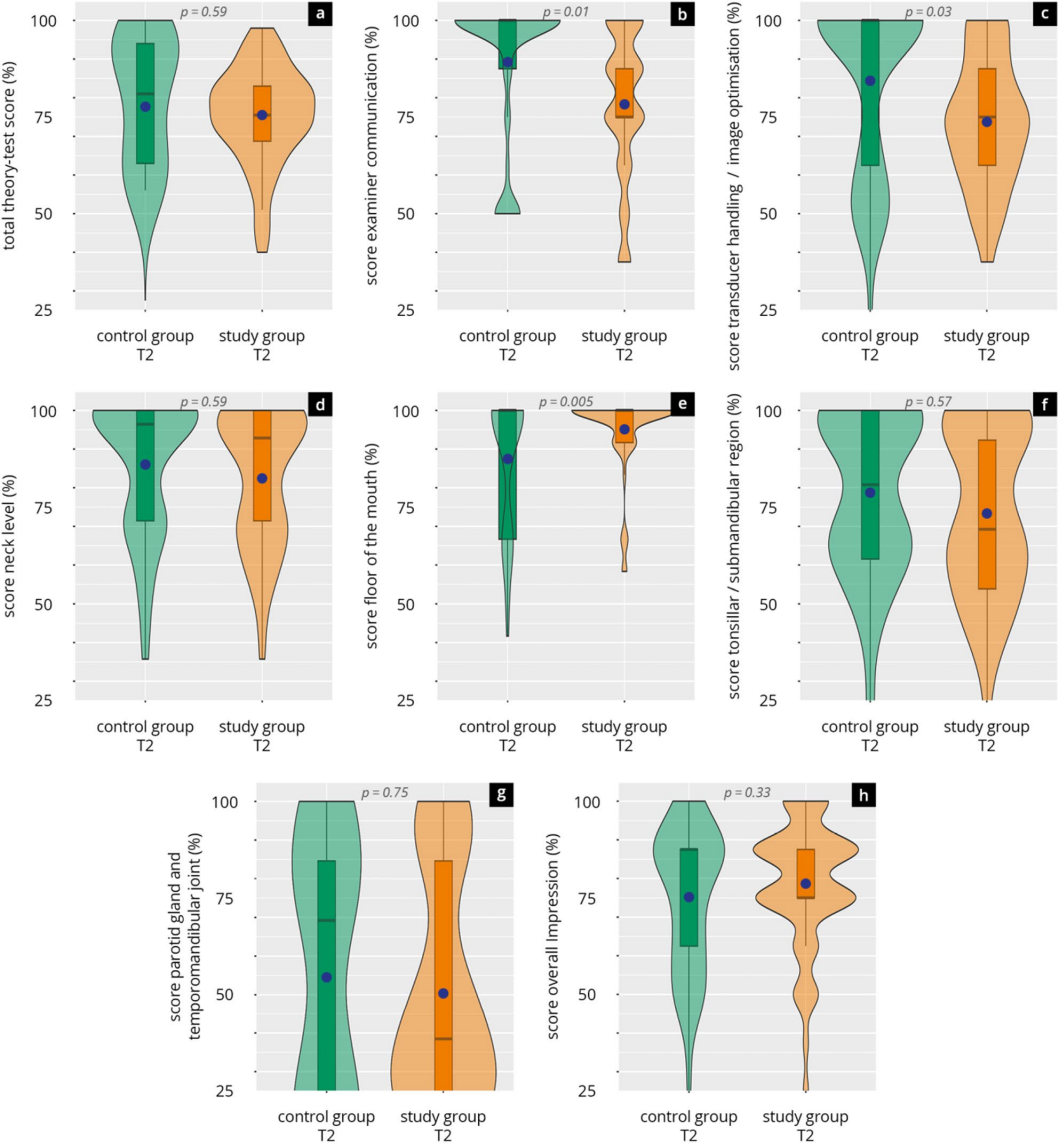
Practical examination (DOPS) results in the study group (students) and the control group (physicians) at T2. Violin plots display the distribution of scores (median [black bar], interquartile range, and mean [blue dot]) for (**a**) total theory test score, **b** examiner communication, **c** transducer handling and image optimisation, **d** neck level, **e** floor of the mouth, (**f**) tonsillar/submandibular region, **g** parotid gland and temporomandibular joint, and (**h**) overall impression. Significance levels of group comparisons are indicated

The results of the DOPS assessment at T2 are shown in Fig. 4 and Supplement 8. The total score across all assessed domains in the student group averaged 75.3 ± 12.8. Among the subdomains, the highest mean score was achieved in the “floor of the mouth” examination (95.3 ± 10.5%), followed by “neck level” (82.5 ± 17.4%) and “examiner communication” (77.0 ± 21.3%). Slightly lower scores were observed in “tonsillar/submandibular region” (73.5 ± 22.2%) and “transducer handling and image optimisation” (73.7 ± 16.9%). The lowest performance was documented in the “parotid gland and temporomandibular joint” module (50.3 ± 30.1%).

The physician reference group achieved a comparable overall DOPS score (77.5 ± 20.9%) to the student group, with no significant difference (*p* = 0.59). In terms of specific domains, physicians achieved significantly higher scores in “examiner communication” (*p* = 0.01) and “transducer handling and image optimisation” (*p* = 0.03). Students performed significantly better in the “floor of the mouth” module (*p* = 0.005). No statistically significant differences were observed in the remaining modules.

### Results of the subjective assessment of competencies (secondary endpoints)

#### Interest and motivation

A detailed overview of temporal trends in interest and motivation is provided in Supplement 9. Throughout the assessment period, students reported the highest levels of interest in radiographic diagnostics (T1: 6.18 ± 0.92) and oral surgery (T2: 5.92 ± 1.32). Among imaging modalities, ultrasound diagnostics also showed notably high interest at T2 (6.11 ± 0.79), with a significant increase compared to T1 (*p* = 0.0004). Regarding motivation, the highest scores were consistently observed for ultrasound diagnostics (T2: 6.32 ± 0.82) and oral surgery (T2: 6.05 ± 1.10).

#### Self-assessment

**Fig. 5:**
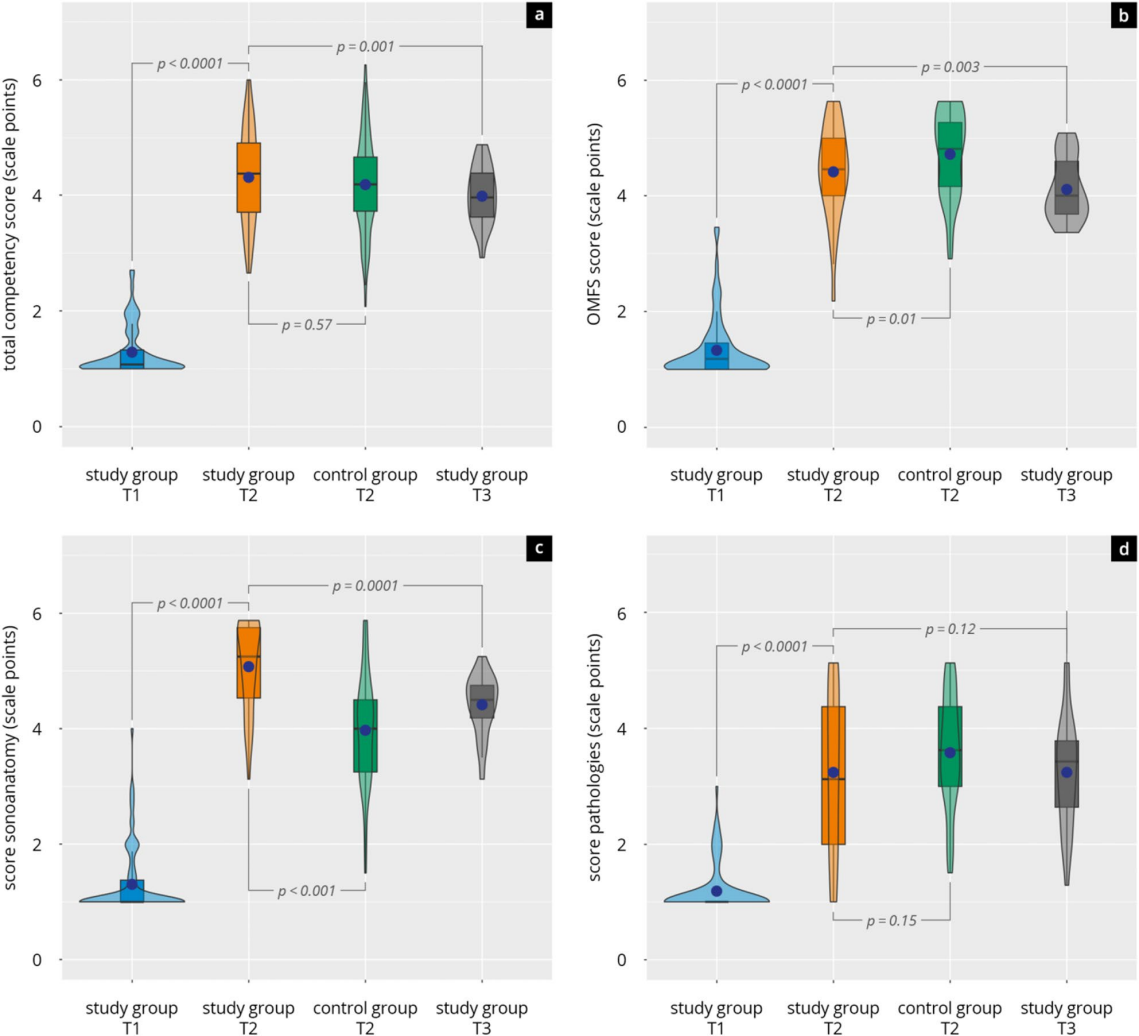
Competency scores in the study group (students) and the control group (physicians) across time points T1, T2, and T3. Violin plots illustrate the distribution of scores (median [black bar], interquartile range, and mean [blue dot]) for (**a**) total competency score, **b** oral and maxillofacial surgery (OMFS) score, **c** sonoanatomy score, and (**d**) pathology score. Significance levels of group comparisons are indicated

The results of all main and sub-items of the assessed subjective competencies are presented in Supplement 10 and Fig. 5.

Across all measured domains, participants reported a marked increase in their self-perceived competencies between T1 and T2. The overall subjective competence score improved significantly (T1: 1.3 ± 0.4, T2: 4.3 ± 0.8; *p* < 0.0001), corresponding to a considerable effect size (d = -4.43). At follow-up (T3), scores remained higher than T1 (4.0 ± 0.5, *p* < 0.001), with a statistically significant but smaller decrease compared to T2 (*p* = 0.001, d = -0.87).

Subdomain analyses supported these findings. In the maxillofacial-surgery ultrasound domain, self-assessments rose from 1.3 ± 0.4 at T1 to 4.4 ± 0.8 at T2 (*p* < 0.0001, d = -4.58) and remained largely stable at T3 (4.1 ± 0.5). The sonoanatomy domain showed the most substantial absolute improvement, increasing from T1 (1.3 ± 0.6) to T2 (5.1 ± 0.8; *p* < 0.0001, d = -5.02), with sustained values at T3 (4.4 ± 0.5; *p* = 0.0001, d = -1.30).

In contrast, while self-perceived competence in the assessment of pathologies also increased significantly from baseline (1.2 ± 0.4) to T2 (3.3 ± 1.5, *p* < 0.0001, d = -1.79), this domain remained lower in absolute terms. It showed only minimal changes at follow-up (T3: 3.2 ± 1.0, *p* = 0.12). Participants reported a marked increase in their self-perceived ability to guide and communicate with patients during sonographic procedures, with mean ratings rising from 1.8 ± 1.3 at T1 to 5.5 ± 1.1 at T2. This significant improvement (Δ = +3.67, *p* < 0.0001, d = − 4.18) remained stable at T3 (5.1 ± 1.1).

In terms of associated imaging modalities, modest increases were also observed in perceived competence related to MRI, CT, and radiographic diagnostics. Contrast PET remained the lowest-rated modality throughout, with only minimal gains.

At T2, the dental students’ overall self-assessment of subjective competencies was comparable to that of the reference group of physicians (*p* = 0.57). In the domain of sonoanatomy, students rated their competencies significantly higher than the physicians (*p* < 0.001, d = − 1.23), while the physicians rated themselves significantly higher in CT (*p* < 0.001, d = 1.24), PET (*p* < 0.001, d = 1.32), and MRI (*p* < 0.001, d = 0.72). Self-assessments in pathology-related competencies showed no significant differences between groups (*p* = 0.15). Students reported higher confidence in radiographic diagnostics than the reference group (*p* = 0.03, d = − 0.39).

### Results regarding the curriculum and teaching materials (secondary endpoints)

**Fig. 6:**
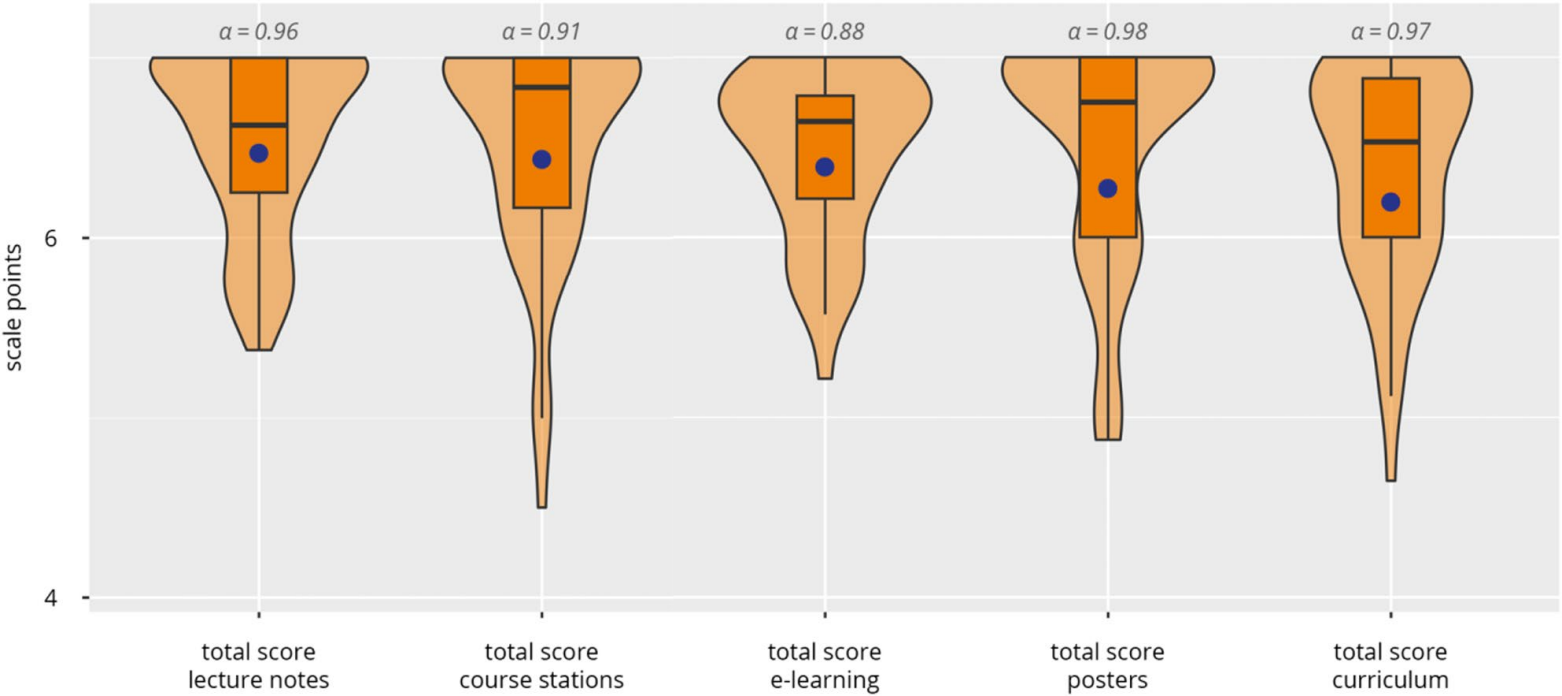
Subjective evaluation of the curriculum’s components. Violin plots show the distribution of total scores (median [black bar], interquartile range, and mean [blue dot]) for lecture notes, course stations, e-learning, posters, and the overall curriculum. Internal consistency for each component is indicated by Cronbach’s α

**Fig. 7:**
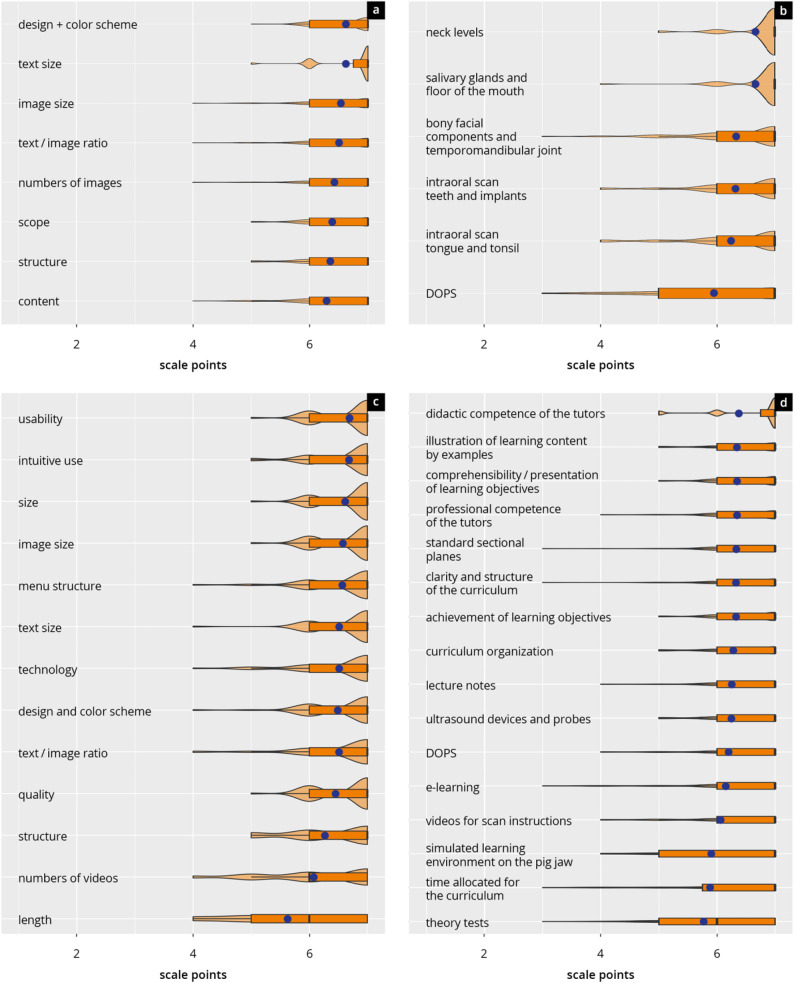
Subitems of the main evaluation scores. Violin plots show the distribution of ratings (median [black bar], interquartile range, and mean [blue dot]) for (**a**) lecture notes, **b** course stations, **c** e-learning, and (**d**) components of the curriculum. Panel (**d**) comprises multiple curricular subitems, including didactic competence of tutors, clarity and structure of the curriculum, comprehensibility of learning objectives, lecture notes, ultrasound devices and probes, instructional videos, simulated learning environment, theory tests, and DOPS. Higher values indicate stronger agreement or satisfaction

Participants provided consistently high ratings across all evaluated components of the curriculum (see Figs. 6 and 7 and Supplement 11).

The e-learning module received particularly high ratings, with an overall mean of 6.4 ± 0.7. Participants especially appreciated the technical stability, visual design, and intuitive usability of the e-learning modules, with several subcomponents, such as menu navigation, text readability, and layout, achieving scores above 6.4. While the general structure and presentation of the learning videos were positively evaluated (mean = 6.3 ± 1.0), ratings for the extent of actual video use (5.6 ± 1.2) showed slightly more variability.

The printed lecture notes were also evaluated favourably, with a mean score of 6.5 ± 0.9. Participants rated both the visual and structural features—such as clarity of organisation, image-to-text ratio, and overall design—consistently above 6.3. Similarly, the educational posters achieved positive overall evaluations (6.3 ± 1.2). Participants valued the clarity, the comprehensibility, the amount of content, and the visual layout, with mean ratings for individual items ranging from 6.0 to 6.4.

The practical course stations were likewise well received, with an overall mean of 6.4 ± 1.0. The stations on neck levels and salivary glands/floor of the mouth received the highest scores (both means = 6.7), while the station focusing on bony facial structures and the temporomandibular joint was rated somewhat lower (mean = 6.0).

General course feedback also demonstrated high levels of satisfaction (6.2 ± 1.7). Participants gave top ratings for course organisation, clarity of learning objectives, and both the didactic and subject-matter competence of the tutors and the DOPS, with mean scores consistently around 6.2 to 6.4. Theoretical components such as the theory tests (5.8 ± 1.4) and the teaching of pathological findings (5.0 ± 1.7), as well as simulation-based components like the porcine jaw model (5.9 ± 1.5), received comparatively lower, yet still moderate to good ratings.

### Results of students’ attitudes toward ultrasound education and teaching methods (secondary endpoints)

Across all three time points (T1–T3), students consistently expressed high levels of agreement regarding the importance of ultrasound education and the value of the teaching formats employed (see Supplement 12). Both theoretical and practical competencies in ultrasonography were rated as essential to be acquired during undergraduate training, with mean values remaining above 5.7 across time. The view that ultrasonography represents a fundamental skill in oral and maxillofacial surgery showed a statistically significant increase from T1 (5.2 ± 1.4) to T2 (M = 6.0 ± 1.1, *p* = 0.0003) and remained stable at T3 (M = 5.9 ± 1.1). Similarly, support for integrating ultrasonography into mandatory dental curricula strengthened significantly between T1 and T2 (*p* < 0.0001) and maintained a high level of approval thereafter. Blended learning methods were also perceived positively throughout (> 5.5 scale points). Agreement with their educational value and their potential to complement traditional teaching formats increased from T1 to T2 (*p* < 0.05) and remained high at T3.

Concerning curricular implementation, students consistently favoured the integration of ultrasound training during the later stages of their studies, with the median preferred time point for course implementation being the 7th semester at all three time points (T1–T3). Regarding the duration of the curriculum, students recommended a minimum of 10–15 teaching hours for ultrasound training, despite some variation over time (T1–T3).

### Correlations and influencing factors

A strong and statistically significant correlation was found between the objective DOPS total score (excluding the global rating) and the examiner’s overall impression score (*r* = 0.81, *p* < 0.001), indicating a high level of alignment between checklist-based procedural assessment and global expert judgement. Correlational analyses between subjective and objective competence scores revealed weak to moderate associations across time points. At T1, the correlation between subjective self-assessed competence and theory test performance was weak and not statistically significant (*r* = 0.12, *p* = 0.30). At T2, a trend toward a moderate positive correlation was observed between subjective competence ratings and theoretical knowledge (*r* = 0.24, *p* = 0.056), as well as with practical DOPS performance (*r* = 0.21, *p* = 0.114). The correlation between theoretical and practical performance at T2 reached nearly statistical significance (*r* = 0.26, *p* = 0.050). At follow-up (T3), correlations between subjective and objective outcomes remained weak and non-significant. Participants who reported engaging with the e-learning platform tended to perform better in the DOPS assessments (mean = 78.5%) compared to those who did not (mean = 74.7%). However, this difference did not reach statistical significance (*p* = 0.34; d = -0.294). To assess potential attrition bias, T2 performance was compared between students who completed the T3 follow-up (*n* = 21) and those who did not (*n* = 43). No significant differences were found in theory test scores (*p* = 0.51), overall subjective competence ratings (*p* = 0.78), or DOPS total scores (*p* = 0.72).

## Discussion

### Educational relevance of ultrasonography in dentistry

Ultrasonography has become increasingly important in dental and maxillofacial diagnostics [6–13, 16, 28, 45, 68]. However, formal training opportunities for dental students remain scarce [22], particularly when compared to general sonography training for other medical professionals [36–42] and medical students [21, 30–35]. This discrepancy points to systemic limitations within dental education, notably the inadequate incorporation of novel diagnostic technologies, insufficient investment in faculty training, and the absence of unified standards across institutions [23]. Thus, this study addresses a critical gap in dental education by demonstrating the feasibility and potential benefits of implementing a structured head and neck ultrasound curriculum for dental students, along with preliminary validation approaches in alignment with national learning objectives for diagnostic imaging.

### Curriculum design

To our knowledge, this study is the first to integrate Kern’s Six-Step curriculum design with the ICAP (Interactive, Constructive, Active, Passive) framework in dental education. This combination provided a systematic, needs-based development process (Kern’s steps) while embedding evidence-based active learning principles (ICAP), representing a novel approach in the dental curriculum context [23, 29, 47]. The resulting blended learning format—comprising video e-learning modules, lecture notes, anatomy posters, and peer-assisted hands-on training, proved to be highly effective. Students demonstrated significant gains in knowledge and an adequate practical skills level after the course, alongside very positive feedback and satisfaction with the course itself. Importantly, benchmarking against a physician reference group provided an external standard for validation: while physicians scored higher on theoretical knowledge, dental students achieved comparable results in the structured DOPS assessment, indicating that core practical competencies can be acquired effectively even by novice learners.

These outcomes from a blended learning-based curriculum align with broader findings in medical education, indicating that blended approaches can yield equal or superior outcomes compared to traditional teaching alone [69]. The high student satisfaction in this program further underscores the value of this multimodal strategy, consistent with reports that learners appreciate the flexibility and depth that blended curricula provide [59, 63]. In sum, an ICAP-informed, blended curriculum represents a pedagogical innovation that not only engages dental students in active learning but also demonstrates validity through reference group comparison, thereby filling an important gap in modern dental training.

### Learning outcomes

Ultrasound training has been rarely incorporated into dentistry, making this dataset a significant contribution. Prior reports were limited to isolated initiatives, such as Kondrashova et al. (2017), who integrated ultrasonography into a dental anatomy course and reported positive student perceptions [22]. However, such initiatives are scarce, and ultrasonography has not been routinely taught in dental schools. By contrast, in undergraduate medicine, ultrasonography has become increasingly mainstream, with numerous programs demonstrating feasibility and benefit [21]. The results show that dental students can acquire ultrasound knowledge and skills at levels comparable to medical trainees [21, 32], indicating that ultrasound education is transferable to dentistry. The comparability of our outcomes to those reported in medical education supports recent calls to integrate emerging diagnostic technologies into dental curricula [23].

Theoretical knowledge improved markedly. This gain reflects both the effectiveness of our teaching materials and possibly a testing effect from repeated assessment. Even though students already possessed a solid foundation in head and neck anatomy, their ultrasound-specific knowledge increased significantly. This finding is consistent with prior work showing that ultrasonography deepens understanding and retention of anatomical structures [24]. Some knowledge decay occurred by the three-month follow-up, consistent with the forgetting curve described in education research [70], but scores remained well above baseline, demonstrating partial long-term retention [71]. This partial sustainability of knowledge underscores the benefit of our curriculum, while also highlighting the need for continued engagement. In line with test-enhanced learning principles, scheduled review sessions or online cases could help stabilise knowledge over time [55]. Importantly, completion of all e-learning modules correlated with higher exam performance, validating the active-learning components of our ICAP-based design. In future iterations, the incorporation of spaced repetition and test-enhanced learning strategies could help stabilise knowledge retention over time.

Alongside knowledge gains, the curriculum led to substantial improvements in practical ultrasound skills. At T2, students achieved an average Direct Observation of Procedural Skills (DOPS) score of approximately 75%, closely comparable to the physician reference group [41]. This demonstrates that dental novices can rapidly acquire core sonographic techniques through structured, peer-assisted training. Similar rapid gains have been observed in other learner groups, such as medical students and residents [40, 72].

Students performed particularly well in simpler tasks such as imaging cervical lymph nodes and thyroid sweeps, but struggled with more complex regions such as the parotid gland and temporomandibular joint, where scores averaged only about 50%. These differences likely reflect both the technical difficulty of these regions and limited emphasis in the e-learning modules. Comparable findings have been reported elsewhere, with novices struggling more in complex head and neck regions [41, 73]. Targeted refinements, including additional practice, advanced simulation, or exposure to pathological cases, could raise competence in these areas. Notably, the skills assessments showed strong agreement between checklist scores and global impressions, supporting the reliability of the evaluation.

Students’ self-confidence increased substantially and remained elevated at follow-up, with many rating their competence nearly equivalent to that of physicians. However, objective results indicated that while students approached physician-level performance in basic tasks, they lagged in theoretical knowledge and recognition of pathology. This discrepancy reflects the common phenomenon of novice overestimation [74]. While strong self-efficacy is a valuable outcome, it underscores the importance of objective assessment and supervised clinical practice to ensure accurate calibration between confidence and competence.

The moderate decline in test scores between posttest and follow-up highlights the importance of longitudinal integration. One-time workshops are insufficient for durable mastery, a conclusion supported by prior studies in ultrasound education [75]. A spiral curriculum, in which ultrasound content is revisited at multiple points across training, could help reinforce skills. Options include refresher sessions, integration into clinical rotations such as oral surgery, or advanced elective modules for interested students. Embedding ultrasound competency into graduation requirements could further encourage sustained practice and retention [39, 70].

Benchmarking against physicians provided valuable validation. For foundational scanning tasks, students performed nearly at physician levels, demonstrating the effectiveness of the training. However, domain-specific differences emerged. Physicians achieved higher scores in “examiner communication” and “transducer handling,” which likely reflect their greater clinical experience and routine in patient interaction and instrument use. Conversely, students performed better in the “floor of the mouth” examination. This may be explained by the fact that this particular scan – often referred to as the “Mickey Mouse” view –became especially memorable and popular among students. These findings suggest that domain-specific familiarity and learning emphasis may influence practical performance as much as prior clinical experience.

However, physicians outperformed students in theoretical knowledge and pathology recognition, which reflects the role of clinical experience. This indicates that while short-term curricula can impart technical competence and basic interpretation skills, diagnostic expertise in pathological cases requires ongoing practice, exposure, and mentorship [76].

These findings also indicate that the curriculum effectively fosters core, dentistry-relevant ultrasound competencies, even though full equivalence with clinically experienced physicians cannot be assumed. Rather than a limitation, this distinction underscores the curriculum’s strength in providing focused, high-quality training for the foundational aspects of head and neck ultrasonography in dental education.

Viewed through the lens of Kirkpatrick’s model, the curriculum achieved success on multiple levels [48, 61]. At Level 1 (Reaction), student satisfaction was extremely high, with mean ratings above six out of seven, and strong endorsement for continuing ultrasound training. At Level 2 (Learning), clear objective gains in knowledge, skills, and confidence were observed. At Level 3 (Behaviour), students reported greater willingness to apply ultrasonography clinically and described new diagnostic considerations arising during their clinical work. Although these findings are based on self-reported perceptions rather than observed behaviour, they indicate a positive attitudinal shift that may facilitate future clinical application.For Kirkpatrick’s Level 4 (Results), patient outcomes were not assessed, but near-physician levels of competence in some tasks imply potential for improved care once these skills are applied in clinical practice.

### Strengths and limitations

Methodologically, the study had several strengths. By following Kern’s systematic design process [50], and embedding established frameworks such as ICAP [47]. and Kirkpatrick [48], we ensured a strong theoretical foundation. The quasi-experimental pre/post design with benchmarking against physicians strengthened internal validity, while assessment tools demonstrated excellent reliability and strong validity evidence. Importantly, the interdisciplinary collaboration in developing and implementing the curriculum, combined with the joint evaluation of the assessment formats and alignment with national learning objectives, represented a particular strength by ensuring both content validity and practical relevance. Adherence to STROBE guidelines further enhanced rigour [49]. Together, these elements support confidence that our findings accurately reflect the impact of the curriculum and provide a replicable template for other institutions.

Nevertheless, limitations must be acknowledged. The high attrition rate, with only one-third of participants completing the three-month follow-up, poses a serious limitation to the validity and generalizability of the long-term retention results. The single-centre, voluntary design limits generalisability, as participants may have been more motivated than average. The absence of a randomised student control group means that improvements cannot be attributed with absolute certainty to the curriculum, although pre-post gains and benchmarking strongly suggest this. The small overlap of physicians completing both tests (*n* = 14) limits comparability across assessments. Actual clinical utilisation and patient outcomes were not assessed, and a cost-effectiveness analysis was not conducted to measure costs associated with equipment acquisition, faculty training time and other expense and effort variables that often influence curricular adoption. Finally, assessments occurred in simulated rather than live clinical settings, so translation to practice remains to be demonstrated.

### Future directions

Future directions include expanding the program into the core dental curriculum. A dual approach, combining compulsory foundational instruction with advanced elective opportunities—potentially including pathological cases and clinical training in oral and maxillofacial surgery—could maximise both the breadth and depth of ultrasound education. Multi-institution collaborations would broaden reach and permit larger-scale evaluation. Possible approaches include integrating the three-month follow-up (T3) into mandatory coursework or scheduled curricular activities, offering participation incentives, and using digital reminder systems to encourage continued engagement. Interprofessional training involving dentistry, oral and maxillofacial surgery, radiology, and otolaryngology could enhance teamwork and enrich the learning experience [39]. For example, mixed workshops with dentists and medical students, or joint training sessions bringing together dentists and ENT residents, could foster interdisciplinary knowledge exchange and strengthen teamwork skills. Pedagogically, refinements should target weaker areas such as pathology recognition and complex anatomical regions, potentially using advanced simulators or digital repositories [77]. Incorporating spaced retrieval practice, interactive case discussions, and new technologies such as AI-assisted ultrasound tutors could support long-term retention [78].

## Conclusion

In conclusion, the positive results of this study lay the groundwork for making head and neck ultrasonography a staple of dental education. By integrating modern imaging training into the dental curriculum, future dentists can be prepared for a more comprehensive diagnostic role and encouraged to embrace new technologies. The success of the blended learning, ICAP-informed approach provides a model that can be replicated and expanded. With further refinement and broader adoption, ultrasound education in dentistry has the potential to enhance diagnostic competencies, improve interdisciplinary collaboration, and ultimately benefit patient care through more timely and accurate management of head and neck conditions. The journey begun with this pilot will continue as our team and other investigators carry this initiative forward, building on the foundation established, and striving for even greater educational and clinical outcomes.

## Supplementary Information

Supplementary Material 1.

Supplementary Material 2.

Supplementary Material 3.

Supplementary Material 4.

Supplementary Material 5.

Supplementary Material 6.

Supplementary Material 7.

Supplementary Material 8.

Supplementary Material 9.

Supplementary Material 10.

Supplementary Material 11.

Supplementary Material 12.

## Data Availability

Data cannot be shared publicly because of institutional and national data policy restrictions imposed by the Ethics committee since the data contain potentially identifying study participants’ information. Data are available upon request from the Johannes Gutenberg University Mainz Medical Center (contact via weimer@uni-mainz.de) for researchers who meet the criteria for access to confidential data (please provide the manuscript title with your inquiry).
