# Supplementary material for: Bridging a curriculum gap: a structured model for integrating head and neck ultrasound training into undergraduate dental education
Source: BMC Med Educ. 2026 Jan 7;26:145. doi: 10.1186/s12909-025-08521-9 (PMC12849422; doi:10.1186/s12909-025-08521-9)
Supplement: Supplementary file 1 — Supplementary Material 1. [file 12909_2025_8521_MOESM1_ESM.pdf]

Supplement 4 DOPS Ultrasonograpy Dentistry

Task:

Perform an orientational head and neck ultrasound examination on a patient.

Guide the patient appropriately through the examination.

Display the following orientation planes and identify the anatomical structures you know/see:

1. Neck level (transition level 3-4 transverse)
2. Floor of the mouth transverse
3. Tonsillar/submandibular region transverse
4. Parotid gland + temporomandibular joint transverse

DOPS Evaluation Sheet

Examiner: **Student ID:**

Patient Guidance/Communication				Points (max. 8)
**It is pointed out**				
	**Correct**	**After prompt**	**Not at all**	
▪ "Please extend your head/tilt to the side"	**2** ◻	**1** ◻	**0** ◻	
▪ Ensure adequate undressing or exposure of the neck + Warns about ultrasound gel	**2** ◻	**1** ◻	**0** ◻	
▪ Place towels and put on gloves	**2** ◻	**1** ◻	**0** ◻	
▪ "Relax your neck" + warns about light pressure during examination	**2** ◻	**1** ◻	**0** ◻	

Transducer Handling and Image Optimization	Points (max. 8)
**Orientation**	
▪ Correct, or immediately self-checked via image movement/disconnection	**2** ◻
▪ Corrected after initial difficulties/upon prompt	**1** ◻
▪ Correct orientation found only with manual assistance	**0** ◻
**Positioning**	
▪ Correct or immediately transferred from another plane	**2** ◻
▪ Corrected after initial difficulties/upon prompt	**1** ◻
▪ Correct position found only with manual assistance	**0** ◻
**Coupling/Transducer Handling**	
▪ Transducer well coupled with sufficient gel, continuous pressure, good handling	**2** ◻
▪ Corrected after initial difficulties/upon prompt	**1** ◻
▪ No pressure and/or no gel + uncontrolled pressure and/or probe half in air	**0** ◻
**Adequate Magnification/Image Optimization/Device Operation**	
▪ Independently and adequately set with appropriate image quality (gain, depth, frequency, focus), left hand on device, also during examt	**2** ◻
▪ Corrected after initial difficulties/upon prompt/forgets optimization during exam, left hand not on device	**1** ◻
▪ No adequate image setting despite prompt, manual assistance necessary	**0** ◻

Adjustment of Orientation Planes	Points (max. 16)

Orientation Plane Neck Level

▪ Orientation plane as complete as possible, good pace	4 □
▪ Verbal help needed for adjustment or very slow pace	2 □
▪ Manual help for adjustment necessary	0 □

Correct identification and naming of structures:

Common carotid artery	0,5 □	Internal jugular vein	0,5 □	Thyroid gland	0,5 □	Sternocleidomastoid muscle	0,5 □
Omohyoid muscle	0,5 □	Trachea	0,5 □				

Orientation Plane Floor of the Mouth

▪ Orientation plane as complete as possible, good pace	4 □
▪ Verbal help needed for adjustment or very slow pace	2 □
▪ Manual help for adjustment necessary	0 □

Correct identification and naming of structures:

Digastric muscle	0,5 □	Mylohyoid muscle	0,5 □	Tongue	0,5 □	Sublingual gland	0,5 □

Orientation Plane Tonsillar/Submandibular Region

▪ Orientation plane as complete as possible, good pace	4 □
▪ Verbal help needed for adjustment or very slow pace	2 □
▪ Manual help for adjustment necessary	0 □

Correct identification and naming of structures:

Tonsils	0,5 □	Submandibular gland	0,5 □	Tongue	0,5 □	Facial vein/artery	0,5 □
Mylohyoid muscle	0,5 □						

Orientation Plane Parotid Gland + Temporomandibular Joint

▪ Orientation plane as complete as possible, good pace	4 □
▪ Verbal help needed for adjustment or very slow pace	2 □
▪ Manual help for adjustment necessary	0 □

Correct identification and naming of structures:

Articular capsule	0,5 □	Mandibular head	0,5 □	Mandibular ramus	0,5 □	Parotid gland	0,5 □

Overall Impression	Points (max. 8)
**I rate the overall impression with (1 to 8 points) (please circle):**<br>1 – 2 – 3 – 4 – 5 – 6 – 7 – 8	

Total points: /
