# Supplementary material for: Bridging a curriculum gap: a structured model for integrating head and neck ultrasound training into undergraduate dental education
Source: BMC Med Educ. 2026 Jan 7;26:145. doi: 10.1186/s12909-025-08521-9 (PMC12849422; doi:10.1186/s12909-025-08521-9)
Supplement: Supplementary file 2 — Supplementary Material 2. [file 12909_2025_8521_MOESM2_ESM.pdf]

Supplement 2: original evaluation instruments

Evaluation Form – Pre-course (T1)

Code: ________________

1. Baseline

1.1 Which degree program are you enrolled in? Human Medicine ☐ Dentistry ☐

1.2 Which semester are you currently in? _____

1.3 Please indicate your gender: m ☐ f ☐ d ☐

1.4 Please state your age: _____

1.5 Do you have prior training in (dental) medicine? Yes ☐ No ☐

1.6 Have you successfully completed the following courses?

	Yes	No
1.6.1 Anatomy	☐	☐
1.6.2 Radiology	☐	☐
1.6.3 Surgical Course 1	☐	☐
1.6.4 Surgical Course 2	☐	☐
1.6.5 Lecture: ENT for Dental Students	☐	☐

2. Pre-course competencies

2.1 Have you previously attended one or more ultrasound courses? Yes ☐ No ☐

2.1.1 If yes, which courses: ____

2.1.2 If yes, what was the duration? _________________h

2.1.3 If yes, did the course cover head and neck sonography? Yes ☐ No ☐

2.1.4 If yes, what was the duration?_________________h

2.2 How many head and neck sonographies have you independently performed?

____________ times

2.3 Do you have experience or prior knowledge in interpreting:

	**Not at all** 1						**Very good** 7
2.3.1 Magnetic Resonance Imaging (MRI)	☐	☐	☐	☐	☐	☐	☐
2.3.2 X-ray diagnostics	☐	☐	☐	☐	☐	☐	☐
2.3.3 Positron Emission Tomography (PET)	☐	☐	☐	☐	☐	☐	☐
2.3.4 Computed Tomography (CT)	☐	☐	☐	☐	☐	☐	☐
2.3.5 Ultrasound	☐	☐	☐	☐	☐	☐	☐

3 Interest and Motivation

3.3 How interested are you in the following medical fields?

	Not at all 1						Very high 7
3.1.1 Oral and Maxillofacial Surgery	☐	☐	☐	☐	☐	☐	☐
3.1.2 Oral Surgery	☐	☐	☐	☐	☐	☐	☐
3.1.3 Otorhinolaryngology (ENT)	☐	☐	☐	☐	☐	☐	☐

3.2 How interested are you in the following imaging techniques?

	Not at all 1						Very high 7
3.2.1 Magnetic Resonance Imaging (MRI)	☐	☐	☐	☐	☐	☐	☐
3.2.2 X-ray diagnostics	☐	☐	☐	☐	☐	☐	☐
3.2.3 Positron Emission Tomography (PET)	☐	☐	☐	☐	☐	☐	☐
3.2.4 Computed Tomography (CT)	☐	☐	☐	☐	☐	☐	☐
3.2.5 Ultrasound							

3.3 The course motivates me to engage with clinical cases in the following areas:

	Not at all 1						Very high 7
3.3.1 Oral and Maxillofacial Surgery	☐	☐	☐	☐	☐	☐	☐
3.3.2 Oral Surgery	☐	☐	☐	☐	☐	☐	☐
3.3.3 Otorhinolaryngology (ENT)	☐	☐	☐	☐	☐	☐	☐

3.4 To what extent do you agree with the following statements?

	Not at all 1						Completely 7
3.4.1 My motivation for participating in the course is very high	☐	☐	☐	☐	☐	☐	☐
3.4.2 The course motivates me to engage with ultrasound diagnostics	☐	☐	☐	☐	☐	☐	☐

4. Subjective Self-Assessment

4.1 How do you currently assess your knowledge regarding OMFS-specific ultrasound, with regard to…

	very low 1						Very high 7
4.1.1 …theoretical knowledge	☐	☐	☐	☐	☐	☐	☐
4.1.2…device operation	☐	☐	☐	☐	☐	☐	☐
4.1.3 …probe handling	☐	☐	☐	☐	☐	☐	☐
4.1.4 …spatial orientation	☐	☐	☐	☐	☐	☐	☐
4.1.5 …sonoanatomical assignment	☐	☐	☐	☐	☐	☐	☐
4.1.6 …structure visualization	☐	☐	☐	☐	☐	☐	☐
4.1.7 …structure assessment	☐	☐	☐	☐	☐	☐	☐
4.1.8 …patient guidance	☐	☐	☐	☐	☐	☐	☐
4.1.9 …safety aspects in ultrasound	☐	☐	☐	☐	☐	☐	☐
4.1.10 …sonographic recognition of pathologies	☐	☐	☐	☐	☐	☐	☐
4.1.11…sonographic assessment of pathologies	☐	☐	☐	☐	☐	☐	☐

4.2 How confident are you currently in basic sonographic depiction (normal findings/sonoanatomy)

	Very unsure 1						Very sure 7
4.2.1 of the floor of the mouth	☐	☐	☐	☐	☐	☐	☐
4.2.2 of the neck levels/soft tissues	☐	☐	☐	☐	☐	☐	☐
4.2.3 of the submandibular space + tonsils	☐	☐	☐	☐	☐	☐	☐
4.2.4 of the parotid gland	☐	☐	☐	☐	☐	☐	☐
4.2.5 of intraoral scan – focus on periodontium	☐	☐	☐	☐	☐	☐	☐
4.2.6 of the temporomandibular joint + masticatory muscles	☐	☐	☐	☐	☐	☐	☐
4.2.7 of the bony landmarks + facial soft tissues	☐	☐	☐	☐	☐	☐	☐
4.2.8 of intraoral scan – focus on tongue and tonsils	☐	☐	☐	☐	☐	☐	☐

4.3 How confident are you currently in basic sonographic assessment of pathologies…

	Very unsure 1						Very sure 7
4.3.1 of the floor of the mouth	☐	☐	☐	☐	☐	☐	☐
4.3.2 of the neck levels/soft tissues	☐	☐	☐	☐	☐	☐	☐
4.3.3 of the submandibular space + tonsils	☐	☐	☐	☐	☐	☐	☐
4.3.4 of the parotid gland	☐	☐	☐	☐	☐	☐	☐
4.3.5 of intraoral scan – focus on periodontium	☐	☐	☐	☐	☐	☐	☐
4.3.6 of the temporomandibular joint + masticatory muscles	☐	☐	☐	☐	☐	☐	☐
4.3.7 of the bony landmarks + facial soft tissues	☐	☐	☐	☐	☐	☐	☐
4.3.8 of intraoral scan – focus on tongue and tonsils	☐	☐	☐	☐	☐	☐	☐

5. Competencies in Associated Areas

5.1 How do you currently assess your competencies regarding the following imaging techniques?

	Very low 1						Very high 7
5.1.1 Magnetic Resonance Imaging (MRI)	☐	☐	☐	☐	☐	☐	☐
5.1.2 X-ray diagnostics	☐	☐	☐	☐	☐	☐	☐
5.1.3 Positron Emission Tomography (PET)	☐	☐	☐	☐	☐	☐	☐
5.1.4 Computed Tomography (CT)	☐	☐	☐	☐	☐	☐	☐

6. General Attitudes Towards (Ultrasound) Teaching and Methods

6.1 To what extent do you agree with the following statements?

	Not at all 1						Completely 7
6.1.1 Ultrasound is an essential basic skill in oral and maxillofacial medicine	☐	☐	☐	☐	☐	☐	☐
6.1.2 General ultrasound competencies should already be acquired during medical studies	☐	☐	☐	☐	☐	☐	☐
6.1.3 Theoretical ultrasound competencies should already be acquired during medical studies	☐	☐	☐	☐	☐	☐	☐
6.1.4 Practical ultrasound competencies should already be acquired during medical studies	☐	☐	☐	☐	☐	☐	☐
6.1.5 Ultrasound training should be integrated into the mandatory curriculum	☐	☐	☐	☐	☐	☐	☐
6.1.6 Ultrasound training should be integrated as an elective/voluntary course	☐	☐	☐	☐	☐	☐	☐
6.1.7 Digital teaching methods (blended learning) are a valuable addition to ultrasound education	☐	☐	☐	☐	☐	☐	☐
6.1.8 Digital teaching methods (blended learning) should be increasingly integrated into teaching	☐	☐	☐	☐	☐	☐	☐
6.1.9 Innovative teaching concepts (blended learning) can strengthen my interest in a particular medical specialty	☐	☐	☐	☐	☐	☐	☐
6.1.10 The choice of future specialization is significantly influenced by the quality of teaching in a given field	☐	☐	☐	☐	☐	☐	☐

6.2 At what point should an ultrasound course be integrated into the curriculum, in your opinion? _________Semester

6.3 What should be the time allocation for ultrasound training within dental studies?
____________h

Evaluation Form Post-course (T2)

Code: ____________________

1. Prior Experience

1.1 After receiving the preparatory materials, were you able to independently gain practical ultrasound experience? Yes ☐ No ☐

1.2 How many head and neck sonographies did you perform independently after receiving the preparatory materials and before the start of the course? ____________ times

Alternatively, how much time did you spend practicing independently before the course?
____________ min

2. Use of Learning Media

2.1 Did you use the provided learning platform "Moodle" to prepare for this ultrasound course? Yes ☐ No ☐

2.2 If yes, how much time did you spend using this platform? (approximate hours)
____

2.3 Did you use the provided "hardcopy lecture notes" to prepare for this ultrasound course?
☐ Yes ☐ No

2.4 If yes, how much time did you spend using this? (approximate hours)_____________________

2.5 Which chapters of the learning media did you read through completely in advance?

- ☐ Basics
- ☐ Floor of the mouth
- ☐ Neck levels
- ☐ Submandibular space + tonsils
- ☐ Parotid gland
- ☐ Intraoral scan of teeth/implants
- ☐ Temporomandibular joint + masticatory muscles
- ☐ Bony landmarks
- ☐ Intraoral scan of tongue and tonsils

3. Evaluation of Learning Materials

3.1. Moodle-E-learning

		Not at all 1						Completely 7
3.1.1	The technology worked well.	☐	☐	☐	☐	☐	☐	☐
3.1.2	The menu structure was appealing.	☐	☐	☐	☐	☐	☐	☐
3.1.3	I watched the videos	☐	☐	☐	☐	☐	☐	☐
3.1.4	The length of the videos was appropriate	☐	☐	☐	☐	☐	☐	☐
3.1.5	The technical quality of the videos (e.g., sound/image) was appropriate	☐	☐	☐	☐	☐	☐	☐
3.1.6	The total number of videos was appropriate.	☐	☐	☐	☐	☐	☐	☐
3.1.7	The structure of the individual videos appealed to me.	☐	☐	☐	☐	☐	☐	☐
3.1.8	The font size of the text was appropriate.	☐	☐	☐	☐	☐	☐	☐
3.1.9	The image size was appropriate.	☐	☐	☐	☐	☐	☐	☐
3.1.10	The video size was appropriate.	☐	☐	☐	☐	☐	☐	☐
3.1.11	The text/image ratio was appropriate	☐	☐	☐	☐	☐	☐	☐
3.1.12	The design and color scheme were appealing	☐	☐	☐	☐	☐	☐	☐
3.1.13	The Moodle e-learning was user-friendly	☐	☐	☐	☐	☐	☐	☐
3.1.14	The operation of the Moodle e-learning was intuitive	☐	☐	☐	☐	☐	☐	☐
3.1.15	Overall, I rate the Moodle e-learning	☐	☐	☐	☐	☐	☐	☐

3.2.1 For Moodle e-learning, I have the following points of praise, criticism, and suggestions for improvement (free text):

3.2 Evaluation of the hardcopy lecture notes

		Not at all 1						Completely 7
3.2.1	The structure and organization appealed to me.	☐	☐	☐	☐	☐	☐	☐
3.2.2	The scope of the individual chapters was appropriate.	☐	☐	☐	☐	☐	☐	☐
3.2.3	The content was conveyed in an understandable way.	☐	☐	☐	☐	☐	☐	☐
3.2.4	The font size was appropriate.	☐	☐	☐	☐	☐	☐	☐
3.2.5	The image size was appropriate.	☐	☐	☐	☐	☐	☐	☐
3.2.6	The number of images was appropriate.	☐	☐	☐	☐	☐	☐	☐
3.2.7	The text/image ratio was appropriate.	☐	☐	☐	☐	☐	☐	☐
3.2.8	The design and color scheme were appealing.	☐	☐	☐	☐	☐	☐	☐
3.2.9	Overall, I rate the lecture notes:	☐	☐	☐	☐	☐	☐	☐

3.2.10 For the hardcopy lecture notes, I have the following points of praise, criticism, and suggestions for improvement (free text):

3.3 Evaluation of Educational Posters (used during the course)

		Not at all 1						Completely 7
3.3.1	The structure and organization of the posters appealed to me.	☐	☐	☐	☐	☐	☐	☐
3.3.2	The scope of the individual posters was appropriate.	☐	☐	☐	☐	☐	☐	☐
3.3.3	The content was conveyed in an understandable way.	☐	☐	☐	☐	☐	☐	☐
3.3.4	The font size was appropriate.	☐	☐	☐	☐	☐	☐	☐
3.3.5	The image size was appropriate.	☐	☐	☐	☐	☐	☐	☐
3.3.6	The number of images was appropriate.	☐	☐	☐	☐	☐	☐	☐
3.3.7	The text/image ratio was appropriate.	☐	☐	☐	☐	☐	☐	☐
3.3.8	The design and color scheme were appealing.	☐	☐	☐	☐	☐	☐	☐
3.3.9.	Overall, I rate the posters:	☐	☐	☐	☐	☐	☐	☐

3.4 Evaluation of Course Stations

How did you like the stations?

		Very dissatisfied 1						Very satisfied 7
3.4.1	Neck levels	☐	☐	☐	☐	☐	☐	☐
3.4.2	Salivary glands and floor of the mouth	☐	☐	☐	☐	☐	☐	☐
3.4.3	Bony facial components and temporomandibular joint	☐	☐	☐	☐	☐	☐	☐
3.4.4	Intraoral scan teeth and implants	☐	☐	☐	☐	☐	☐	☐
3.4.5	Intraoral scan tongue and tonsil	☐	☐	☐	☐	☐	☐	☐
3.5.5	DOPS	☐	☐	☐	☐	☐	☐	☐

3.4.6 For the course stations, I have the following points of praise, criticism, and suggestions for improvement (free text):

4 Course Feedback

How satisfied are you with…	Very dissatisfied 1						Very satisfied 7
4.1 The clarity and structure of the course concept	☐	☐	☐	☐	☐	☐	☐
4.2 The comprehensibility/presentation of learning objectives	☐	☐	☐	☐	☐	☐	☐
4.3 Achievement of learning objectives	☐	☐	☐	☐	☐	☐	☐
4.4 Illustration of learning content by examples	☐	☐	☐	☐	☐	☐	☐
4.5 Course organization	☐	☐	☐	☐	☐	☐	☐
4.6 The time allocated for the course and the balance between theory and practice	☐	☐	☐	☐	☐	☐	☐
4.7 The ultrasound lecturre notes	☐	☐	☐	☐	☐	☐	☐
4.8 The Moodle e-learning	☐	☐	☐	☐	☐	☐	☐
4.9 The standard planes as orientation aids.	☐	☐	☐	☐	☐	☐	☐
4.10 The videos for scan instructions	☐	☐	☐	☐	☐	☐	☐
4.11 The pathologies covered	☐	☐	☐	☐	☐	☐	☐
4.12 The professional competence of the tutors	☐	☐	☐	☐	☐	☐	☐
4.13 The didactic competence of the tutors	☐	☐	☐	☐	☐	☐	☐
4.14 The ultrasound devices and probes	☐	☐	☐	☐	☐	☐	☐
4.15 The simulated learning environment on the pig jaw	☐	☐	☐	☐	☐	☐	☐
4.16 The theory tests	☐	☐	☐	☐	☐	☐	☐
4.17 The practical DOPS	☐	☐	☐	☐	☐	☐	☐

4.18 Would you like to give us any individual feedback about the course? (free text)

5 Interest and Motivation

5.1 How interested are you in the following medical fields after the course?

	Not at all 1						Very high 7
5.1.1 Oral and Maxillofacial Surgery	☐	☐	☐	☐	☐	☐	☐
5.1.2 Oral Surgery	☐	☐	☐	☐	☐	☐	☐
5.1.3 Otorhinolaryngology (ENT)	☐	☐	☐	☐	☐	☐	☐

5.2 How interested are you in the following imaging techniques after the course?

	Not at all 1						Very high 7
5.2.1 Magnetic Resonance Imaging (MRI)	☐	☐	☐	☐	☐	☐	☐
5.2.2 X-ray diagnostics	☐	☐	☐	☐	☐	☐	☐
5.2.3 Positron Emission Tomography (PET)	☐	☐	☐	☐	☐	☐	☐
5.2.4 Computed Tomography (CT)	☐	☐	☐	☐	☐	☐	☐
5.2.5 Ultrasound	☐	☐	☐	☐	☐	☐	☐

5.3 The course motivates me to engage with clinical cases in the following areas...

	Very low 1						Very high 7
5.3.1 Oral and Maxillofacial Surgery	☐	☐	☐	☐	☐	☐	☐
5.3.2 Oral Surgery	☐	☐	☐	☐	☐	☐	☐
5.3.3 Otorhinolaryngology (ENT)	☐	☐	☐	☐	☐	☐	☐

5.4 To what extent do you agree with the following statement?

	Very low 1						Very high 7
The course motivates me to engage with ultrasound diagnostics	☐	☐	☐	☐	☐	☐	☐

6 Subjective Self-Assessment

6.1. How do you currently assess your knowledge regarding OMFS-specific ultrasound, with regard to...

	very low 1						Very high 7
6.1.1 …theoretical knowledge	☐	☐	☐	☐	☐	☐	☐
6.1.2…device operation	☐	☐	☐	☐	☐	☐	☐
6.1.3 …probe handling	☐	☐	☐	☐	☐	☐	☐
6.1.4 …spatial orientation	☐	☐	☐	☐	☐	☐	☐
6.1.5 …sonoanatomical assignment	☐	☐	☐	☐	☐	☐	☐
6.1.6 …structure visualization	☐	☐	☐	☐	☐	☐	☐
6.1.7 …structure assessment	☐	☐	☐	☐	☐	☐	☐
6.1.8 …patient guidance	☐	☐	☐	☐	☐	☐	☐
6.1.9 …safety aspects in ultrasound	☐	☐	☐	☐	☐	☐	☐
6.1.10 …sonographic recognition of pathologies	☐	☐	☐	☐	☐	☐	☐
6.1.11…sonographic assessment of pathologies	☐	☐	☐	☐	☐	☐	☐

6.2. How confident are you currently in basic sonographic depiction (normal findings/sonoanatomy)

	Very unsure 1						Very sure 7
6.2.1 of the floor of the mouth	☐	☐	☐	☐	☐	☐	☐
6.2.2 of the neck levels/soft tissues	☐	☐	☐	☐	☐	☐	☐
6.2.3 of the submandibular space + tonsils	☐	☐	☐	☐	☐	☐	☐
6.2.4 of the parotid gland	☐	☐	☐	☐	☐	☐	☐
6.2.5 of intraoral scan – focus on periodontium	☐	☐	☐	☐	☐	☐	☐
6.2.6 of the temporomandibular joint + masticatory muscles	☐	☐	☐	☐	☐	☐	☐
6.2.7 of the bony landmarks + facial soft tissues	☐	☐	☐	☐	☐	☐	☐
6.2.8 of intraoral scan – focus on tongue and tonsils	☐	☐	☐	☐	☐	☐	☐

6.3. How confident are you currently in basic sonographic assessment of pathologies…

	Very unsure 1						Very sure 7
6.3.1 of the floor of the mouth	☐	☐	☐	☐	☐	☐	☐
6.3.2 of the neck levels/soft tissues	☐	☐	☐	☐	☐	☐	☐
6.3.3 of the submandibular space + tonsils	☐	☐	☐	☐	☐	☐	☐
6.3.4 of the parotid gland	☐	☐	☐	☐	☐	☐	☐
6.3.5 of intraoral scan – focus on periodontium	☐	☐	☐	☐	☐	☐	☐
6.3.6 of the temporomandibular joint + masticatory muscles	☐	☐	☐	☐	☐	☐	☐
6.3.7 of the bony landmarks + facial soft tissues	☐	☐	☐	☐	☐	☐	☐
6.3.8 of intraoral scan – focus on tongue and tonsils	☐	☐	☐	☐	☐	☐	☐

7. Competencies in Associated Areas

How do you currently assess your competencies regarding the following imaging techniques?

	Very low 1						Very high 7
7.1.1 Magnetic Resonance Imaging (MRI)	☐	☐	☐	☐	☐	☐	☐
7.1.2 X-ray diagnostics	☐	☐	☐	☐	☐	☐	☐
7.1.3 Positron Emission Tomography (PET)	☐	☐	☐	☐	☐	☐	☐
7.1.4 Computed Tomography (CT)	☐	☐	☐	☐	☐	☐	☐

8. General Attitudes Towards (Ultrasound) Teaching and Methods

8.1 To what extent do you agree with the following statements?

	Not at all 1						Completely 7
8.1.1 Ultrasound is an essential basic skill in oral and maxillofacial medicine	☐	☐	☐	☐	☐	☐	☐
8.1.2 General ultrasound competencies should already be acquired during medical studies	☐	☐	☐	☐	☐	☐	☐
8.1.3 Theoretical ultrasound competencies should already be acquired during medical studies	☐	☐	☐	☐	☐	☐	☐
8.1.4 Practical ultrasound competencies should already be acquired during medical studies	☐	☐	☐	☐	☐	☐	☐
8.1.5 Ultrasound training should be integrated into the mandatory curriculum	☐	☐	☐	☐	☐	☐	☐
8.1.6 Ultrasound training should be integrated as an elective/voluntary course	☐	☐	☐	☐	☐	☐	☐
8.1.7 Digital teaching methods (blended learning) are a valuable addition to ultrasound education	☐	☐	☐	☐	☐	☐	☐
8.1.8 Digital teaching methods (blended learning) should be increasingly integrated into teaching	☐	☐	☐	☐	☐	☐	☐
8.1.9 Innovative teaching concepts (blended learning) can strengthen my interest in a particular medical specialty	☐	☐	☐	☐	☐	☐	☐
8.1.10 The choice of future specialization is significantly influenced by the quality of teaching in a given field	☐	☐	☐	☐	☐	☐	☐

8.2 At what point should an ultrasound course be integrated into the curriculum, in your opinion? _________Semester

8.3 What should be the time allocation for ultrasound training within dental studies? ____________h

8.4. Would you like to give us any additional feedback? (free text)

Evaluation Form Follow up T3

Code: _______________

1. General

1.1 Did you participate in the course "Ultrasound in Dentistry"?

☐ Yes ☐ No

1.2 After the course, were you able to independently gain practical ultrasound experience? Yes☐ No☐

1.3 How many head and neck sonographies did you perform independently after the course? ____________ times

2 Interest and Motivation

2.1. How interested are you in the following medical fields?

	Not at all 1						Very high 7
2.1.1 Oral and Maxillofacial Surgery	☐	☐	☐	☐	☐	☐	☐
2.1.2 Oral Surgery	☐	☐	☐	☐	☐	☐	☐
2.1.3 Otorhinolaryngology (ENT)	☐	☐	☐	☐	☐	☐	☐

2.2. How interested are you in the following imaging techniques after the course?

	Not at all 1						Very high 7
2.2.1 Magnetic Resonance Imaging (MRI)	☐	☐	☐	☐	☐	☐	☐
2.2.2 X-ray diagnostics	☐	☐	☐	☐	☐	☐	☐
2.2.3 Positron Emission Tomography (PET)	☐	☐	☐	☐	☐	☐	☐
2.2.4 Computed Tomography (CT)	☐	☐	☐	☐	☐	☐	☐
2.2.5 Ultrasound							

2.3. The course motivates me to engage with clinical cases in the following areas:

	Not at all 1						Very high 7
2.3.1 Oral and Maxillofacial Surgery	☐	☐	☐	☐	☐	☐	☐
2.3.2 Oral Surgery	☐	☐	☐	☐	☐	☐	☐
2.3.3 Otorhinolaryngology (ENT)	☐	☐	☐	☐	☐	☐	☐

2.4. To what extent do you agree with the following statements?

	Not at all 1						Completely 7
2.4.1 The course motivates me to engage with ultrasound diagnostics	☐	☐	☐	☐	☐	☐	☐

3. Subjective Self-Assessment

3.1. How do you currently assess your knowledge regarding OMFS-specific ultrasound, with regard to…

	very low 1						Very high 7
3.1.1 …theoretical knowledge	☐	☐	☐	☐	☐	☐	☐
3.1.2…device operation	☐	☐	☐	☐	☐	☐	☐
3.1.3 …probe handling	☐	☐	☐	☐	☐	☐	☐
3.1.4 …spatial orientation	☐	☐	☐	☐	☐	☐	☐
3.1.5 …sonoanatomical assignment	☐	☐	☐	☐	☐	☐	☐
3.1.6 …structure visualization	☐	☐	☐	☐	☐	☐	☐
3.1.7 …structure assessment	☐	☐	☐	☐	☐	☐	☐
3.1.8 …patient guidance	☐	☐	☐	☐	☐	☐	☐
3.1.9 …safety aspects in ultrasound	☐	☐	☐	☐	☐	☐	☐
3.1.10 …sonographic recognition of pathologies	☐	☐	☐	☐	☐	☐	☐
3.1.11…sonographic assessment of pathologies	☐	☐	☐	☐	☐	☐	☐

3.2. How confident are you currently in basic sonographic depiction (normal findings/sonoanatomy)?

	Very unsure 1						Very sure 7
3.2.1 of the floor of the mouth	☐	☐	☐	☐	☐	☐	☐
3.2.2 of the neck levels/soft tissues	☐	☐	☐	☐	☐	☐	☐
3.2.3 of the submandibular space + tonsils	☐	☐	☐	☐	☐	☐	☐
3.2.4 of the parotid gland	☐	☐	☐	☐	☐	☐	☐
3.2.5 of intraoral scan – focus on periodontium	☐	☐	☐	☐	☐	☐	☐
3.2.6 of the temporomandibular joint + masticatory muscles	☐	☐	☐	☐	☐	☐	☐
3.2.7 of the bony landmarks + facial soft tissues	☐	☐	☐	☐	☐	☐	☐
3.2.8 of intraoral scan – focus on tongue and tonsils	☐	☐	☐	☐	☐	☐	☐

3.3. How confident are you currently in basic sonographic assessment of pathologies?

	Very unsure 1						Very sure 7
3.3.1 of the floor of the mouth	☐	☐	☐	☐	☐	☐	☐
3.3.2 of the neck levels/soft tissues	☐	☐	☐	☐	☐	☐	☐
3.3.3 of the submandibular space + tonsils	☐	☐	☐	☐	☐	☐	☐
3.3.4 of the parotid gland	☐	☐	☐	☐	☐	☐	☐
3.3.5 of intraoral scan – focus on periodontium	☐	☐	☐	☐	☐	☐	☐
3.3.6 of the temporomandibular joint + masticatory muscles	☐	☐	☐	☐	☐	☐	☐
3.3.7 of the bony landmarks + facial soft tissues	☐	☐	☐	☐	☐	☐	☐
3.3.8 of intraoral scan – focus on tongue and tonsils	☐	☐	☐	☐	☐	☐	☐
	☐	☐	☐	☐	☐	☐	☐

4. Competencies in Associated Areas

4.1 How do you currently assess your competencies regarding the following imaging techniques?

	Very low 1						Very high 7
4.1.1 Magnetic Resonance Imaging (MRI)	☐	☐	☐	☐	☐	☐	☐
4.1.2 X-ray diagnostics	☐	☐	☐	☐	☐	☐	☐
4.1.3 Positron Emission Tomography (PET)	☐	☐	☐	☐	☐	☐	☐
4.1.4 Computed Tomography (CT)	☐	☐	☐	☐	☐	☐	☐

5. General Attitudes Towards (Ultrasound) Teaching and Methods

5.1 To what extent do you agree with the following statements?

	Not at all 1						Completely 7
5.1.1 Ultrasound is an essential basic skill in oral and maxillofacial medicine	☐	☐	☐	☐	☐	☐	☐
5.1.2 General ultrasound competencies should already be acquired during medical studies	☐	☐	☐	☐	☐	☐	☐
5.1.3 Theoretical ultrasound competencies should already be acquired during medical studies	☐	☐	☐	☐	☐	☐	☐
5.1.4 Practical ultrasound competencies should already be acquired during medical studies	☐	☐	☐	☐	☐	☐	☐
5.1.5 Ultrasound training should be integrated into the mandatory curriculum	☐	☐	☐	☐	☐	☐	☐
5.1.6 Ultrasound training should be integrated as an elective/voluntary course	☐	☐	☐	☐	☐	☐	☐
5.1.7 Digital teaching methods (blended learning) are a valuable addition to ultrasound education	☐	☐	☐	☐	☐	☐	☐
5.1.8 Digital teaching methods (blended learning) should be increasingly integrated into teaching	☐	☐	☐	☐	☐	☐	☐
5.1.9 Innovative teaching concepts (blended learning) can strengthen my interest in a particular medical specialty	☐	☐	☐	☐	☐	☐	☐
5.1.10 The choice of future specialization is significantly influenced by the quality of teaching in a given field	☐	☐	☐	☐	☐	☐	☐

5.2 At what point should an ultrasound course be integrated into the curriculum, in your opinion? _________Semester

5.3 What should be the time allocation for ultrasound training within dental studies?
____________h
