# Supplementary material for: Bridging a curriculum gap: a structured model for integrating head and neck ultrasound training into undergraduate dental education
Source: BMC Med Educ. 2026 Jan 7;26:145. doi: 10.1186/s12909-025-08521-9 (PMC12849422; doi:10.1186/s12909-025-08521-9)
Supplement: Supplementary file 3 — Supplementary Material 3. [file 12909_2025_8521_MOESM3_ESM.pdf]

Supplement 1 Theoretical and practical learning objectives of the head and neck ultrasound curriculum

Theoretical learning objectives	Practical learning objectives
• Explain safety aspects of head and neck ultrasound.<br>• Describe the physics of image formation, artifacts, devices, probe maneuvers, and imaging modes.<br>• Identify the design features of ultrasound machines and explain knobology.<br>• Differentiate transducer types and their appropriate use.<br>• Summarize technical and equipment basics relevant for dental/maxillofacial sonography.<br>• Explain ultrasound orientation views/sections.<br>• Identify and describe anatomical head and neck structures in sonographic images (floor of mouth, cervical levels, submandibular space and tonsils, parotid gland, teeth/implants, Temporomandibular Joint [TMJ], masticatory muscles, osseous facial structures, tongue/tonsils).<br>• Outline options for documenting ultrasound findings.<br>• Recognize and classify common pathologies (abscess, fracture, benign/malignant lymph nodes, peri-implantitis, masseter hypertrophy, salivary gland stones, cysts, TMJ effusion).	• Perform correct machine setup (transducer selection, presets, image optimization).<br>• Demonstrate proper transducer handling (holding, movements, stabilization, connection).<br>• Label, and draw ultrasound orientation views.<br>• Adjust head and neck ultrasound orientation views during scanning.<br>• Identify cervical lymph node levels and major vascular landmarks in practice.<br>• Visualize and assess salivary glands in live scans.<br>• Demonstrate intraoral ultrasound examinations of tongue, tonsils, teeth and dental implants and visualize peri-implant tissues<br>• Examine the floor of the mouth, tongue, and oropharyngeal structures systematically.<br>• Recognize sonoanatomical landmarks of the TMJ and masticatory muscles.
