# Supplementary material for: Bridging a curriculum gap: a structured model for integrating head and neck ultrasound training into undergraduate dental education
Source: BMC Med Educ. 2026 Jan 7;26:145. doi: 10.1186/s12909-025-08521-9 (PMC12849422; doi:10.1186/s12909-025-08521-9)
Supplement: Supplementary file 4 — Supplementary Material 4. [file 12909_2025_8521_MOESM4_ESM.pdf]

Supplement 6 Baselinecharacteristic of the reference group

**Question**	**Category/Scale**	**Valid percentage/statistics**
number of participants		141
Study Program	Human medicine	82.4%
	Dental medicine	1.1%
	Both	16.5%
Year of training	Mean ± SD	2.8 ± 2.9
Gender	diverse	2.2%
	male	50.0%
	female	47.8%
Age	Mean ± SD	30.9 ± 5.2
	Median [Min–Max]	30.0 [24–55]
Current Level of Training	Resident	93.5%
	Specialist	4.3%
	Consultant	2.2%
Have you already participated in one or more ultrasound courses?	yes	40.2%
	no	59.8%
If yes, what was the duration (hours)?	Mean ± SD	16.5 ± 7.7
	Median [Min–Max]	16.0 [1–40]
If yes, did the course cover head and neck sonography?	yes	83.3%
	no	16.7%
If yes, what was the duration (hours)?	Mean ± SD	15.1 ± 6.4
	Median [Min–Max]	16.0 [1–36]
How many head and neck sonographic examinations have you already performed independently?	Mean ± SD	167.4 ± 244.0
	Median [Min–Max]	67.5 [1–1200]
Test participation		
Participation in DOPS	yes	54
	no	87
Participation in theory test	yes	98
	no	43
both	yes	14
	no	127
