# Supplementary material for: Bridging a curriculum gap: a structured model for integrating head and neck ultrasound training into undergraduate dental education
Source: BMC Med Educ. 2026 Jan 7;26:145. doi: 10.1186/s12909-025-08521-9 (PMC12849422; doi:10.1186/s12909-025-08521-9)
Supplement: Supplementary file 5 — Supplementary Material 5. [file 12909_2025_8521_MOESM5_ESM.pdf]

Supplement 5 Baselinecharacteristic of the study group

Item	Category/Scale	Valid percentage/statistics
Total number of participants		64
semester	5	5.6%
	6	10.9%
	7	10.9%
	8	23.4%
	9	23.4%
	10	10.9%
	n.A.	4.7%
Gender	male	23.4%
	female	71.9%
	n.A.	4.7%
Age	Mean ± SD	23.9 ± 3.7
prior training in (dental) medicine	yes	25%
	no	75%
Have you successfully completed the following courses?		
– Anatomy	yes	95.3%
	no	4.7%
– Radiology	yes	67.2%
	no	31.8%
– Surgical Course 1	yes	34.4%
	no	63.6%
– Surgical Course 2	yes	12.5%
	no	87.5%
– Lecture: ENT for Dental Students	yes	10.9%
	no	89.1%
Have you previously attended one or more ultrasound courses?	yes	1.6%
	no	98.5%
If yes, what was the duration?		1 participant: 8 hours
If yes, did the course cover head and neck sonography?	yes	1.6%
	no	98.4%
If yes, what was the duration?		1 participant: 1 hour,
How many head and neck sonographies have you independently performed?	Mean ± SD	0 ± 0
Did you use the provided learning platform "Moodle" to prepare for this ultrasound course?	yes	87.3%
	no	12.7%
If yes, how much time did you spend using this platform? (approximate hours)	Mean ± SD	5.0 ± 13.1
Did you use the provided "hardcopy lecture notes" to prepare for this ultrasound course?	yes	88.9%
	no	11.1%
Which chapters of the learning media did you read through completely in advance?		
– Basics	yes	71.2%
	no	28.8%
– Floor of the mouth	yes	91.5%
	no	8.5%
– Neck levels	yes	79.7%
	no	20.3%
– Submandibular space + tonsils	yes	67.8%

	no	32.2%
– Parotid gland	yes	71.2%
	no	28.8%
– Intraoral scan of teeth/implants	yes	52.5%
	no	47.5%
– Temporomandibular joint + masticatory muscles	yes	59.3%
	no	40.7%
– Bony landmarks	yes	45.8%
	no	54.2%
– Intraoral scan of tongue and tonsils	yes	49.2%
	no	50.8%
