# Supplementary material for: Bridging a curriculum gap: a structured model for integrating head and neck ultrasound training into undergraduate dental education
Source: BMC Med Educ. 2026 Jan 7;26:145. doi: 10.1186/s12909-025-08521-9 (PMC12849422; doi:10.1186/s12909-025-08521-9)
Supplement: Supplementary file 6 — Supplementary Material 6. [file 12909_2025_8521_MOESM6_ESM.pdf]

Supplement 12 Results of students' general attitudes towards ultrasound teaching and methodology across three time points (T1–T3)

Question	T1 Mean± SD; Median [IQR]	T2 Mean ± SD; Median [IQR]	Delta T1-T2 and p-value		T3 Mean ± SD; Median [IQR]	Delta T2-T3 und p-value		p-value Kruskal-Wallis
Ultrasound is an essential basic skill in oral and maxillofacial medicine	5.2 ± 1.4; 5 [4–6]	6.0 ± 1.1; 6 [5–7]	-0.8	< 0.001	5.9 ± 1.1; 6 [5–7]	0.2	0.2	< 0.001
General ultrasound competencies should already be acquired during medical studies	6.5 ± 1.3 ; [5–7]	6.2 ± 1.1; 6 [6–7]	0.6	0.54	6.1 ± 0.9; 6 [5–7]	0.0	1.0	0.06
Theoretical ultrasound competencies should already be acquired during medical studies	5.8 ± 1.3; 6 [5–7]	6.2 ± 1.1; 6.5 [6–7]	-0.4	0.02	6.1 ± 0.9; 6 [5–7]	-0.1	0.68	0.03
Practical ultrasound competencies should already be acquired during medical studies	5.9 ± 1.2; 6 [6–7]	6.4 ± 0.7; 7 [6–7]	-0.5	0.001	6.3 ± 0.9; 7 [6–7]	0.1	0.45	0.02
Ultrasound training should be integrated into the mandatory curriculum	4.8 ± 1.6; 5 [4–6]	5.6 ± 1.4; 6 [5–7]	-0.7	<0.001	5.6 ± 1.4; 6 [5–7]	0.1	0.54	0.002
Ultrasound training should be integrated as an elective/voluntary course	5.6 ± 1.6; 6 [5–7]	5.7 ± 1.7; 6 [5–7]	0.1	0.8	5.8 ± 1.3; 6 [5–7]	0.1	0.75	0.006
Digital teaching methods (blended learning) are a valuable addition to ultrasound education	5.8 ± 1.2; 6 [5–7]	6.2 ± 1.3; 7 [6–7]	-0.4	0.05	6.2 ± 1.1; 7 [6–7]	0.5	0.02	0.007
Digital teaching methods (blended learning) should be increasingly integrated into teaching	5.6 ± 1.5; 6 [4–7]	6.1 ± 1.4; 7 [6–7]	-0.6	0.001	6.1 ± 1.2; 6 [5–7]	0.5	0.02	0.008
Innovative teaching concepts (blended learning) can strengthen my interest in a particular medical specialty	5.7 ± 1.4; 6 [5–7]	5.8 ± 1.3; 6 [5–7]	-0.2	0.45	6.1 ± 1.1; 6 [5–7]	0.4	0.1	0.38
The choice of future specialization is significantly influenced by the quality of teaching in a given field	6.0 ± 1.3; 7 [6–7]	6.2 ± 1.2; 7 [6–7]	-0.3	0.08	5.8 ± 1.7; 6 [5–7]	0.5	0.08	0.35
At what point should an ultrasound course be integrated into the curriculum, in your opinion?	7.1 ± 1.3; 7 [7–8]	6.5 ± 1.5; 7 [6–7]	0.5	0.02	6.4 ± 1.4; 7 [6–7]	0.3	0.29	0.06
What should be the time allocation for ultrasound training within dental studies?	18.0 ± 13.8; 15 [10–20]	14.1 ± 12.8; 10 [6.5–19]	5.1	0.03	17.6 ± 26.2; 10 [2–20]	-5.9	0.23	0.03
