# Supplementary material for: Bridging a curriculum gap: a structured model for integrating head and neck ultrasound training into undergraduate dental education
Source: BMC Med Educ. 2026 Jan 7;26:145. doi: 10.1186/s12909-025-08521-9 (PMC12849422; doi:10.1186/s12909-025-08521-9)
Supplement: Supplementary file 7 — Supplementary Material 7. [file 12909_2025_8521_MOESM7_ESM.pdf]

Supplement 11 Results of ratings across all evaluated components of the curriculum

Complex/Question	Mean ± SD	Median	IQR 1-3
**Overall teaching material score**	6.4 ± 0.8	6.7	6.1¬-7
**Overall e-learning score**	6.4 ± 0.7	6.6	6.2-6.8
The technology worked well.	6.5 ± 1.0	7.0	6–7
The menu structure was appealing.	6.6 ± 0.8	7.0	6–7
I watched the videos	5.3 ± 1.9	6.0	4–7
The length of the videos was appropriate	5.6 ± 1.2	6.0	5–7
The technical quality of the videos (e.g., sound/image) was appropriate	6.4 ± 1.1	7.0	6–7
The total number of videos was appropriate.	6.1 ± 1.1	6.0	5–7
The structure of the individual videos appealed to me.	6.3 ± 1.0	7.0	6–7
The font size of the text was appropriate.	6.5 ± 1.1	7.0	6–7
The image size was appropriate.	6.6 ± 0.9	7.0	6–7
The video size was appropriate.	6.6 ± 0.9	7.0	6–7
The text/image ratio was appropriate	6.5 ± 1.1	7.0	6–7
The design and color scheme were appealing	6.5 ± 1.0	7.0	6–7
The Moodle e-learning was user-friendly	6.7 ± 0.5	7.0	6–7
The operation of the Moodle e-learning was intuitive	6.7 ± 0.6	7.0	6–7
Overall, I rate the Moodle e-learning	6.4 ± 0.6	7.0	6–7
**Overall lecture notes score**	6.5 ± 0.9	6.6	6.6-7
The structure and organization appealed to me.	6.4 ± 1.0	7.0	6–7
The scope of the individual chapters was appropriate.	6.4 ± 1.0	7.0	6–7
The content was conveyed in an understandable way.	6.3 ± 1.0	7.0	6–7
The font size was appropriate.	6.6 ± 0.9	7.0	6–7
The image size was appropriate.	6.5 ± 1.0	7.0	6–7
The number of images was appropriate.	6.4 ± 1.1	7.0	6–7
The text/image ratio was appropriate.	6.5 ± 1.0	7.0	6–7
The design and color scheme were appealing.	6.6 ± 0.9	7.0	6–7
Overall, I rate the lecture notes:	6.4 ± 1.0	7.0	6–7
**Overall poster score**	6.3 ±1.2	6.8	6–7
The structure and organization of the posters appealed to me.	6.2 ± 1.2	6.5	6–7
The scope of the individual posters was appropriate	6.0 ± 1.4	6.0	5–7
The content was conveyed in an understandable way.	6.3 ± 1.3	7.0	6–7
The font size was appropriate.	6.2 ± 1.4	7.0	6–7
The image size was appropriate.	6.4 ± 1.2	7.0	6–7
The number of images was appropriate.	6.2 ± 1.4	7.0	6–7
The text/image ratio was appropriate.	6.4 ± 1.3	7.0	6–7
The design and color scheme were appealing.	6.4 ± 1.2	7.0	6–7
Overall, I rate the posters:	6.2 ± 1.3	7.0	6–7
**Overall course stations score**	6.4 ± 0.9	6.8	6 - 7
neck levels	6.7 ± 1.1	7.0	7–7
salivary glands and floor of the mouth	6.7 ± 1.1	7.0	7–7
bony facial components and temporomandibular joint	6.0 ± 1.4	7.0	5–7
intraoral scan teeth and implants	6.3 ± 1.1	7.0	6–7
intraoral scan tongue and tonsil	6.3 ± 1.3	7.0	6–7
DOPS	6.3 ± 1.3	7.0	6–7
**Overall feedback score**	6.2 ±1.7	6.5	6–7
The clarity and structure of the course concept	6.3 ± 1.4	7	6–7
The comprehensibility/presentation of learning objectives	6.3 ± 1.3	7	6–7
Achievement of learning objectives	6.3 ± 1.3	7	6–7
Illustration of learning content by examples	6.3 ± 1.4	7	6–7
Course organization	6.3 ± 1.4	7	6–7

The time allocated for the course and the balance between theory and practice	5.9 ± 1.5	6	5–7
The ultrasound lecturre notes	6.3 ± 1.4	7	6–7
The Moodle e-learning	6.2 ± 1.2	7	6–7
The standard planes as orientation aids	6.3 ± 1.3	7	6–7
The videos for scan instructions	6.1 ± 1.4	7	6–7
The pathologies covered	5.0 ± 1.7	5	4–6
The professional competence of the tutors	6.3 ± 1.4	7	6–7
The didactic competence of the tutors	6.4 ± 1.4	7	6–7
The ultrasound devices and probes	6.3 ± 1.4	7	6–7
The simulated learning environment on the pig jaw	5.9 ± 1.5	6	5–7
The theory tests	5.8 ± 1.4	6	5–7
The practical DOPS	6.2 ± 1.4	7	6–7
