# Supplementary material for: Bridging a curriculum gap: a structured model for integrating head and neck ultrasound training into undergraduate dental education
Source: BMC Med Educ. 2026 Jan 7;26:145. doi: 10.1186/s12909-025-08521-9 (PMC12849422; doi:10.1186/s12909-025-08521-9)
Supplement: Supplementary file 8 — Supplementary Material 8. [file 12909_2025_8521_MOESM8_ESM.pdf]

Supplement 10 Results of all main and sub-items of the assessed subjective competencies

Variable	T1 Mean ± SD, Median, [IQR]	T2 Mean ± SD, Median, [IQR]	Delta T1-T2	p-value	T3 Mean, SD, Median, IQR)	Delta T2-T3	p-value	Kruskal-Wallis p-value	T2 reference group Mean ± SD, Median, [IQR]	p-value studygroup vs. reference group
**Overall subjective score**	1.3 ± 0.4; 1 [1-1.3]	4.3±0.8; 4.3 [3.7–5]	-3.0	<0.0001	4.0±0.5; 4 [3.6–4.4]	-0.5	0.001	<0.0001	4.9 ± 1.2; 4.8 [3.7 – 4.6]	0.57
**Overall OMFS score (Oral and Maxillofacial Surgery)**	1.3 ± 0.5; 1.1 [1-1.5]	4.4±0.8; 4.5 [4–5]	-3.1	<0.0001	4.1±0.5; 4 [3.7–5]	0.3	0.003	<0.0001	4.0 ± 1.0; 4.0 [4.2 – 5.3]	0.01
theoretical knowledge	1.7 ± 1.0; 1 [1–2]	4.6 ± 1.0; 5 [4–5]	-2.9	< 0.001	4.0 ± 1.0; 4 [3–5]	-0.7	0.003	< 0.001	4.3 ± 1.0; 4.0 [4.0 – 5.0]	
device operation	1.2 ± 0.6; 1 [1–1]	4.7 ± 1.0; 5 [4–5]	-3.5	< 0.001	4.3 ± 0.8; 4 [4–5]	-0.4	0.07	< 0.001	4.1 ± 1.1; 4.0 [3.0 – 5.0]	
probe handling	1.4 ± 0.8; 1 [1–1]	5.0 ± 1.0; 5 [4–6]	-3.6	< 0.001	4.7 ± 0.9; 5 [4–5]	-0.3	0.18	< 0.001	5.1 ± 1.1; 5.0 [4.0 – 6.0]	
spatial orientation	1.5 ± 0.9; 1 [1–2]	4.9 ± 1.0; 5 [4–5]	-3.3	< 0.001	4.2 ± 0.7; 4 [3–5]	-0.9	< 0.001	< 0.001	4.8 ± 1.0; 5.0 [4.0 – 5.0]	
sonoanatomical assignment	1.2 ± 0.6; 1 [1–1]	4.8 ± 1.0; 5 [4–5]	-3.5	< 0.001	4.0 ± 1.0; 4 [3–5]	-0.9	< 0.001	< 0.001	4.7 ± 1.0; 5.0 [4.0 – 5.0]	
structure visualization	1.3 ± 0.7; 1 [1–1]	4.9 ± 1.1; 5 [4–6]	-3.6	< 0.001	4.6 ± 0.8; 5 [4–5]	-0.6	0.007	< 0.001	4.9 ± 1.0; 5.0 [4.0 – 6.0]	
structure assessment	1.3 ± 0.6; 1 [1–1]	4.6 ± 1.2; 5 [4–5]	-3.3	< 0.001	3.8 ± 1.0; 4 [3–5]	-1.2	< 0.001	< 0.001	4.6 ± 1.0; 5.0 [4.0 – 5.0]	
patient guidance	1.8 ± 1.3; 1 [1–2]	5.5 ± 1.1; 6 [5–6]	-3.8	< 0.001	5.1 ± 1.1; 5 [4–6]	-0.7	< 0.001	< 0.001	5.5 ± 1.1; 6.0 [5.0 – 6.0]	

safety aspects in ultrasound	1.3 ± 0.8; 1 [1–1]	4.6 ± 1.5; 5 [3–6]	-3.3	< 0.001	4.6 ± 1.0; 5 [4–5]	-0.02	0.65	< 0.001	5.0 ± 1.4; 5.0 [4.0 – 6.0]	
sonographic recognition of pathologies	1.1 ± 0.4; 1 [1–1]	3.1 ± 1.4; 3 [2–4]	-2.0	< 0.001	3.0 ± 0.9; 3 [2–3]	+0.1	0.62	< 0.001	4.6 ± 1.1; 5.0 [4.0 – 5.0]	
sonographic assessment of pathologies	1.1 ± 0.4; 1 [1–1]	2.8 ± 1.3; 2.5 [2–3.8]	-1.7	< 0.001	2.7 ± 1.0; 2.5 [2–3]	0.0	1.00	< 0.001	4.4 ± 1.1; 4.0 [4.0 – 5.0]	
**Overall sonoanatomy score**	1.3 ± 0.6; 1 [1–1.4]	5.1 ± 0.8; 5.3 [4.5–5.8]	-3.8	<0.001	4.4 ± 0.5; 4.5 [4.2–4.8]	0.7	< 0.001	<0.001	4.0 ± 1.0; 4.0 [3.3 – 4.5]	<0.001
of the floor oft he mouth	1.3 ± 0.7; 1 [1–1]	5.9 ± 0.9; 6 [5–7]	-4.5	< 0.001	5.1 ± 1.1; 5 [4–6]	-0.9	0.003	< 0.001	4.9 ± 1.3; 5.0 [4.0 – 6.0]	
of the neck levels/soft tissues	1.3 ± 0.6; 1 [1–1]	5.3 ± 0.9; 5 [5–6]	-4.0	< 0.001	4.4 ± 1.1; 5 [3–5]	-1.1	< 0.001	< 0.001	5.0 ± 1.2; 5.0 [4.0 – 6.0]	
of the submandibular space + tonsils	1.4 ± 0.7; 1 [1–1]	5.3 ± 1.1; 5 [5–6]	-3.8	< 0.001	4.8 ± 0.7; 5 [4–5]	-0.5	0.04	< 0.001	4.7 ± 1.2; 5.0 [4.0 – 6.0]	
of the parotid gland	1.4 ± 0.8; 1 [1–1]	5.4 ± 1.0; 5 [5–6]	-3.8	< 0.001	4.8 ± 0.6; 5 [4–5]	-0.4	0.18	< 0.001	5.8 ± 1.3; 5.0 [4.0 – 6.0]	
of the intraoral scan – focus on periodontium	1.2 ± 0.7; 1 [1–1]	4.9 ± 1.4; 5 [4–6]	-3.5	< 0.001	4.3 ± 1.1; 5 [4–5]	-0.6	0.02	< 0.001	2.3 ± 1.5; 2.0 [1.0 – 3.0]	
of the temporomandibular joint + masticatory muscles	1.3 ± 0.8; 1 [1–1]	4.9 ± 1.1; 5 [4–6]	-3.5	< 0.001	4.4 ± 0.8; 5 [4–5]	-0.5	0.003	< 0.001	2.9 ± 1.6; 2.5 [1.0 – 4.0]	
of the bony landmarks + facial soft tissues	1.3 ± 0.6; 1 [1–1]	4.9 ± 1.1; 5 [4–6]	-3.6	< 0.001	4.2 ± 0.8; 4 [3–5]	-0.7	0.005	< 0.001	3.8 ± 1.6; 4.0 [2.0 – 5.0]	
of the intraoral scan – focus on tongue and tonsils	1.2 ± 0.6; 1 [1–1]	4.8 ± 1.1; 5 [4–6]	-3.6	< 0.001	4.2 ± 0.8; 4 [3–5]	-0.6	0.003	< 0.001	2.6 ± 1.6; 2.0 [1.0 – 4.0]	
**Overall pathology score**	1.2 ± 0.4; 1 [1–1]	3.3 ± 1.5 ; 3.1 [2–4.4]	-2.1	<0.0001	3.2 ± 1.0; 3.5 [2.6–3.8]	0.1	0.12	<0.0001	3.6 ± 1.0; 3.6 [3.0 – 4.4]	0.15

of the floor oft he mouth	1.2 ± 0.4; 1 [1–1]	3.4 ± 1.6; 3 [2–5]	-2.3	< 0.001	3.5 ± 1.4; 3 [2–5]	+0.3	0.44	< 0.001	4.3 ± 1.3; 5.0 [4.0 – 5.0]	
of the neck levels/soft tissues	1.2 ± 0.4; 1 [1–1]	3.4 ± 1.6; 3 [2–5]	-2.2	< 0.001	3.0 ± 1.4; 3 [1–4]	+0.9	0.05	< 0.001	4.6 ± 1.2; 5.0 [4.0 – 5.0]	
of the submandibular space + tonsils	1.2 ± 0.4; 1 [1–1]	3.2 ± 1.5; 3 [2–4]	-2.2	< 0.001	3.3 ± 1.0; 3 [2–4]	+0.4	0.32	< 0.001	4.2 ± 1.3; 4.0 [3.0 – 5.0]	
of the parotid gland	1.3 ± 0.6; 1 [1–1]	3.3 ± 1.5; 3 [2–5]	-2.0	< 0.001	3.1 ± 0.9; 3 [2–4]	+0.7	0.08	< 0.001	4.8 ± 1.4; 5.0 [4.0 – 6.0]	
of the intraoral scan – focus on periodontium	1.1 ± 0.4; 1 [1–1]	3.5 ± 1.8; 3 [2–5]	-2.2	< 0.001	3.3 ± 1.1; 3 [2–4]	+0.9	0.01	< 0.001	2.1 ± 1.4; 1.5 [1.0 – 3.0]	
of the temporomandibular joint + masticatory muscles	1.2 ± 0.6; 1 [1–1]	3.3 ± 1.6; 3 [2–5]	-2.0	< 0.001	3.2 ± 1.2; 3 [2–4]	+0.5	0.19	< 0.001	2.5 ± 1.5; 2.0 [1.0 – 4.0]	
of the bony landmarks + facial soft tissues	1.2 ± 0.5; 1 [1–1]	3.3 ± 1.5; 3 [2–5]	-2.0	< 0.001	3.1 ± 1.3; 3 [2–4]	+0.3	0.37	< 0.001	3.4 ± 1.6; 3.5 [2.0 – 5.0]	
of the intraoral scan – focus on tongue and tonsils	1.2 ± 0.4; 1 [1–1]	3.2 ± 1.5; 3 [2–5]	-1.9	< 0.001	3.1 ± 1.5; 3 [2–4]	+0.0	1.00	< 0.001	2.6 ± 1.5; 2.0 [1.0 – 4.0]	
**Associated areas**										
Magnetic Resonance Imaging (MRI)	1.9 ± 1.0; 2 [1–2]	2.5 ± 1.3; 2 [1–3]	-0.6	< 0.001	2.9 ± 1.3; 3 [2–4]	+0.1	0.45	< 0.001	3.4 ± 1.2; 3.0 [2.0 – 4.0]	
X-ray diagnostics	3.6 ± 1.8; 4 [1–5]	4.6 ± 1.7; 5 [4–6]	-0.9	< 0.001	5.1 ± 1.1; 5 [4–6]	+0.3	0.19	< 0.001	4.0 ± 1.2; 4.0 [3.0 – 5.0]	
Positron Emission Tomography (PET)	1.2 ± 0.5; 1 [1–1]	1.6 ± 0.9; 1 [1–2]	-0.4	< 0.001	1.7 ± 0.9; 1 [1–2]	+0.1	0.5	0.002	3.2 ± 1.4; 3.0 [2.0 – 4.0]	
Computed Tomography (CT)	2.4 ± 1.5; 2 [1–3]	2.9 ± 1.4; 3 [2–4]	-0.5	0.03	3.4 ± 1.6; 3 [2–5]	+0.1	0.5	0.01	4.5 ± 1.2; 5.0 [4.0 – 5.0]	
