# Supplementary material for: Bridging a curriculum gap: a structured model for integrating head and neck ultrasound training into undergraduate dental education
Source: BMC Med Educ. 2026 Jan 7;26:145. doi: 10.1186/s12909-025-08521-9 (PMC12849422; doi:10.1186/s12909-025-08521-9)
Supplement: Supplementary file 9 — Supplementary Material 9. [file 12909_2025_8521_MOESM9_ESM.pdf]

Supplement 9 Ratings of diagnostic methods, interest and motivation across three time points T1–T3

Question	T1 Mean ± SD, Median, [IQR]	T2 Mean ± SD, Median, [IQR]	T3 Mean ± SD, Median, [IQR]	Kruskal-Wallis p-value
**Oral and Maxillofacial Surgery**	4.7 ± 1.8; 5 [4–6]	5.3 ± 1.5; 6 [4.5–6]	4.7 ± 1.5; 5 [4–6]	0.11
**Oral Surgery**	5.5 ± 1.4; 6 [5–7]	5.9 ± 1.3; 6 [5.5–7]	5.3 ± 1.4; 6 [4.5–6]	0.03
**Otorhinolaryngology (ENT)**	3.2 ± 1.7; 3 [2–4]	4.0 ± 1.8; 4 [3–6]	3.7 ± 1.3; 4 [3–5]	0.04
**Magnetic Resonance Imaging (MRI)**	5.0 ± 1.4; 5 [4–6]	5.2 ± 1.4; 5 [4–6]	4.8 ± 1.1; 5 [4–5]	0.25
**X-ray diagnostics**	6.2 ± 0.9; 6 [6–7]	5.91 ± 1.27; 6 [6–7]	5.7 ± 1.1; 6 [5–6]	0.12
**Positron Emission Tomography (PET)**	3.7 ± 1.8; 3 [2–5]	4.0 ± 1.5; 4 [3–5]	3.3 ± 1.5; 3 [2–4]	0.18
**Computed Tomography (CT)**	5.4 ± 1.4; 6 [4–6]	5.4 ± 1.3; 6 [5–6]	4.7 ± 1.5; 5 [4–6]	0.14
**Ultrasound**	5.4 ± 1.2; 6 [4–6]	6.1 ± 0.8; 6 [6–7]	5.6 ± 0.9; 5 [5–6]	0.0004
**Motivation Oral and Maxillofacial Surgery**	5.9 ± 1.2; 6 [5–7]	6.0 ± 1.1; 6 [5.25–7]	5.4 ± 0.9; 5 [5–6]	0.01
**Motivation Oral Surgery**	6.0 ± 1.0; 6 [5–7]	6.1 ± 1.1; 6 [6–7]	5.4 ± 0.9; 5 [5–6]	0.004
**Motivation Otorhinolaryngology (ENT)**	4.3 ± 1.7; 4 [3–6]	4.7 ± 1.6; 5 [4–6]	4.4 ± 1.1; 4 [4–5]	0.26
**Motivation Ultrasound**	6.0 ± 1.1; 6 [6–7]	6.3 ± 0.8; 6 [6–7]	5.9 ± 1.0; 6 [5–7]	0.06
