# Supplementary material for: Bridging a curriculum gap: a structured model for integrating head and neck ultrasound training into undergraduate dental education
Source: BMC Med Educ. 2026 Jan 7;26:145. doi: 10.1186/s12909-025-08521-9 (PMC12849422; doi:10.1186/s12909-025-08521-9)
Supplement: Supplementary file 11 — Supplementary Material 11. [file 12909_2025_8521_MOESM11_ESM.docx]

**Supplement 7** Results of the theory test at time points T1–T3

| **Competence area** | **T1 Mean ± SD (%)**  **T1 Median**  **(IQR 1–3) (%)** | **T2 Mean ± SD (%)**  **T2 Median**  **(IQR 1–3) (%)** | **T3 Mean ± SD (%)**  **T3 Median**  **(IQR 1–3) (%)** | **p-value Krushk. Test.** | **Delta T1-T2 Mean Diff (%), p, d)** | **Delta T2-T3 Mean Diff (%), p, d)** | **T2 reference group Mean ± SD (%), Median (IQR 1–3) (%)** | **p-value studygroup vs. reference group** |
| --- | --- | --- | --- | --- | --- | --- | --- | --- |
| Anatomical basics | 71.1 ± 20.8;  75.0  [58.3–83.3] | 77.2 ± 15.5  75.0  [75.0–85.4] | 81.5 ± 15.3  83.3  [75.0–91.7] | 0.64 | -4.0, 0.13, -0.4 | -1.5, 0.62, -0.1 | 67.4 ± 17.9, 66.7, [50.0–83.3] | 0.0003 |
| Ultrasound basics | 17.6 ± 16.2  16.0  [4.0–24.0] | 57.4 ± 17.0  60.0  [48.0–68.0] | 51.1 ± 18.8  52.0  [34.0–66.0] | **< 0.001** | -34.6, **< 0.001**, -2.1 | -7.5, 0.02, -0.43 | 75.4 ± 15.5, 80.0, [68.0–88.0] | < 0.0001 |
| Assignment tasks | 52.3 ± 17.1  50.0  [50.0–75.0] | 79.3 ± 20.2  75.0  [75.0–100.0] | 79.4 ± 17.9  75.0  [75.0–100.0] | **< 0.001** | -28.23, **< 0.001**, -1.8 | -5.4, 0.2, -0.3 | 78.3 ± 19.9, 75.0, [62.5–100.0] | 0.76 |
| Normal findings | 0.7 ± 2.4  0.0  [0.0–0.0] | 57.4 ± 16.7  59.3  [44.4–66.7] | 35.1 ± 22.6  33.3  [18.5–50.0] | **< 0.001** | -59.4, **< 0.001**, -5.2 | -26.1, **< 0.001**, -1.3 | 55.4 ± 16.8, 55.6, [40.7–66.7] | 0.47 |
| Pathologies | 1.6 ± 4.7  0.0  [0.0–0.0] | 24.2 ± 19.1  22.7  [9.1–36.4] | 17.4 ± 18.2  9.1  [0.0–31.8] | **< 0.001** | -21.7, 0.0002, -1.3 | -7.5, 0.09, -0.4 | 45.7 ± 20.1, 45.5, [27.3–63.6] | < 0.0001 |
| Total score | 19.5 ± 7.5  18.4  [13.9–24.1] | 56.9 ± 11.9  58.9  [49.4–64.6] | 47.0 ± 13.5  51.9  [35.4–55.7] | **< 0.001** | -36.3, **< 0.001**, -4.1 | -12.8, **< 0.001**, -1.1 | 63.4 ± 12.9, 65.2, [53.2–73.4] | 0.001 |
