# Supplementary material for: Bridging a curriculum gap: a structured model for integrating head and neck ultrasound training into undergraduate dental education
Source: BMC Med Educ. 2026 Jan 7;26:145. doi: 10.1186/s12909-025-08521-9 (PMC12849422; doi:10.1186/s12909-025-08521-9)
Supplement: Supplementary file 12 — Supplementary Material 12. [file 12909_2025_8521_MOESM12_ESM.docx]

**Supplement 8** Results of the DOPS assessment at T2

| **Question** | **Reference group Mean ± SD (%)** | **Reference group Median (%)** | **IQR (1.–3. Quartil) (%)** | **Study group**  **Mean ± SD (%)** | **Study group Median (%)** | **IQR (1.–3. Quartil) (%)** | **p-value** | **Delta Mean**  **(reference group – study group) (%)** |
| --- | --- | --- | --- | --- | --- | --- | --- | --- |
| Examiner communication | 87.5 ± 22.7 | 100.0 | 58.3–100.0 | 77.0 ± 21.3 | 75.0 | 65.6–87.5 | **0.01** | **+10.5** |
| Transducer handling and image optimization | 82.9 ± 25.0 | 100.0 | 68.0–100.0 | 73.7 ± 16.9 | 75.0 | 62.5–87.5 | **0.03** | **+9.2** |
| Cervical levels | 84.5 ± 21.3 | 92.9 | 71.4–100.0 | 82.5 ± 17.4 | 92.9 | 71.4–100.0 | 0.59 | **+2.0** |
| Floor of the mouth | 86.0 ± 21.2 | 100.0 | 89.6–100.0 | 95.3 ± 10.5 | 100.0 | 91.7–100.0 | **0.005** | **-9.3** |
| Tonsillar / submandibular space | 76.5± 24.9 | 69.2 | 53.9–92.3 | 73.5 ± 22.2 | 69.2 | 53.9–92.3 | 0.57 | **+2.9** |
| Parotid gland + temporomandibular joint | 52.6± 35.7 | 53.9 | 23.1–84.6 | 50.3 ± 30.1 | 38.5 | 23.1–84.6 | 0.75 | **+2.4** |
| Overall impression | 75.2 ± 18.4 | 87.5 | 75.0–87.5 | 78.4 ± 16.2 | 75.0 | 75.0–87.5 | 0.33 | **-3.2** |
| Total Score DOPS | 77.5 ± 20.9 | 81.0 | 68.8–83.0 | 75.3 ± 12.8 | 75.5 | 68.8–83.0 | 0.59 | **+2.2** |
| Total Score DOPS without overall impression | 71.5 ± 19.7 | 75.0 | 61.7–75.3 | 68.0 ± 11.7 | 69.0 | 61.7–75.3 | 0.42 | **+3.2** |
| Score practical | 72.9 ± 28.0 | 75.0 | 50.0–87.5 | 70.8 ± 17.2 | 75.0 | 62.5–78.1 | 0.64 | **+2.1** |
| Score demonstration | 62.9 ± 17.2 | 60.0 | 55.0–75.0 | 64.4 ± 12.6 | 65.0 | 55.0–75.0 | 0.66 | **-1.5** |
